# Brain-derived CCR5 Contributes to Neuroprotection and Brain Repair after Experimental Stroke

**DOI:** 10.14336/AD.2020.0406

**Published:** 2021-02-01

**Authors:** Suning Ping, Xuecheng Qiu, Michele Kyle, Li-Ru Zhao

**Affiliations:** Department of Neurosurgery, State University of New York Upstate Medical University, New York, USA

**Keywords:** CCR5, stroke, neuroprotection, neurological deficits, subacute phase

## Abstract

Chemokine (C-C motif) receptor 5 (CCR5) is expressed not only in the immune cells but also in cerebral cells such as neurons, glia, and vascular cells. Stroke triggers high expression of CCR5 in the brain. However, the role of CCR5 in stroke remains unclear. In this study, using bone marrow chimeras we have determined the involvement of brain-derived or bone marrow-derived CCR5 in neuroprotection and brain repair after experimental stroke. CCR5^-/-^ mice that received either wild-type (WT) or CCR5^-/-^ bone marrow transplantation showed larger infarction sizes than the WT mice that received either WT or CCR5^-/-^ bone marrow transplantation in both the acute (48h) and subacute (2 months) phases after cerebral cortical ischemia, suggesting that the lack of CCR5 in the brain leads to severe brain damage after stroke. However, the lack of CCR5 in the bone marrow-derived cells did not affect infarction size. The impairments of somatosensory-motor function and motor coordination were exacerbated in the mice lacking CCR5 in the brain. At 2 months post-stroke, increased degenerative neurons, decreased dendrites and synapses, decreased Iba1^+^ microglia/ macrophages, reduced myelination and CNPase^+^ oligodendrocytes in the peri-infarct cortex were observed in the mice lacking CCR5 in the brain. These pathological changes are significantly correlated with the increased infarction size and exacerbated neurological deficits. These data suggest that brain-derived CCR5 plays a key role in neuroprotection and brain repair in the subacute phase of stroke. This study reveals a novel role of CCR5 in stroke, which sheds new light on post-stroke pathomechanism.

Stroke is a cerebrovascular disease in which brain tissue death and neurological deficits occur from the sudden interruption of blood flow to a specific region of the brain. Based on the pathological characteristics and timing post-stroke, a stroke is generally classified into three clinical phases: the acute phase, subacute phase and chronic phase [1]. The exact duration of each phase is different for individuals according to their age, injury location, the metabolic state of brain and the severity of brain ischemia [1]. Generally, the acute phase is the first 48h after stroke symptom onset, the subacute phase is the period between 48h and 3 months, and the chronic phase starts 3 months after stroke [2-6]. Over the past few decades, major advances have been made in understanding the pathophysiology of stroke, especially in the acute phase, when massive numbers of neurons undergo damage and neuroinflammation is triggered [7]. There has been a growing body of clinical and biomedical research targeting brain repair and functional improvement in the chronic phase, when neurological status becomes relatively stable [8-13]. However, little is known about the pathological changes in the subacute phase of stroke, particularly in the late subacute phase.

Complex biochemical and cellular signaling cascades are involved in brain damage after stroke. C-C chemokine receptor 5 (CCR5) is a G protein-coupled receptor involved in recruiting leukocytes to the site of tissue damage [14]. CCR5 is highly expressed in T cells [15] and macrophages [16] in the immune system. In addition, CCR5 expression is also found in microglia [17], astrocytes [18], neurons [19], cerebral endothelial cells [20, 21] and vascular smooth muscle cells [16] in the central nervous system (CNS). It has been reported that the gene expression levels of CCR5 and its ligands are markedly upregulated 24h and 7 days after cerebral cortical ischemia. Genetic ablation of CCR5 (i.e., CCR5 knockout mice) results in the increase of infarction size and exacerbation of motor functional deficits after experimental stroke [22], demonstrating neuroprotective effects of CCR5 in ischemic brain injury. It remains unclear, however, whether CCR5 is needed for brain repair in the subacute phase.

Despite the permanent brain tissue damage, spontaneous brain repair and functional recovery continually occur from days to several months post-stroke [1]. The mechanism underlying the spontaneous recovery after stroke has not been fully understood. Recent studies in animal models of ischemic stroke [23, 24] have revealed the pivotal role of the infiltrating monocyte-derived macrophages (MDMs) in brain repair. It has been shown that MDMs are required to modulate the local inflammation and promote long-term spontaneous function recovery after stroke [23]. As mentioned earlier, ischemic brain injury triggers upregulation of CCR5 expression in the brain; CCR5 is expressed not only in the recruited macrophages from circulation but also in the cerebral cells (neurons, glia, and vascular cells). It remains unknown whether CCR5 expression in brain resident cells or CCR5 expression in the infiltrating hematogenous cells is critical for neuroprotection and brain repair after ischemic injury. The aim of this study is to address this question by using CCR5 bone marrow chimeras. In this study, we have identified the effects of brain-derived CCR5 or bone marrow-derived CCR5 on neuroprotection in the acute and subacute phases and on brain repair in the subacute phase of experimental stroke.

**Figure 1.**
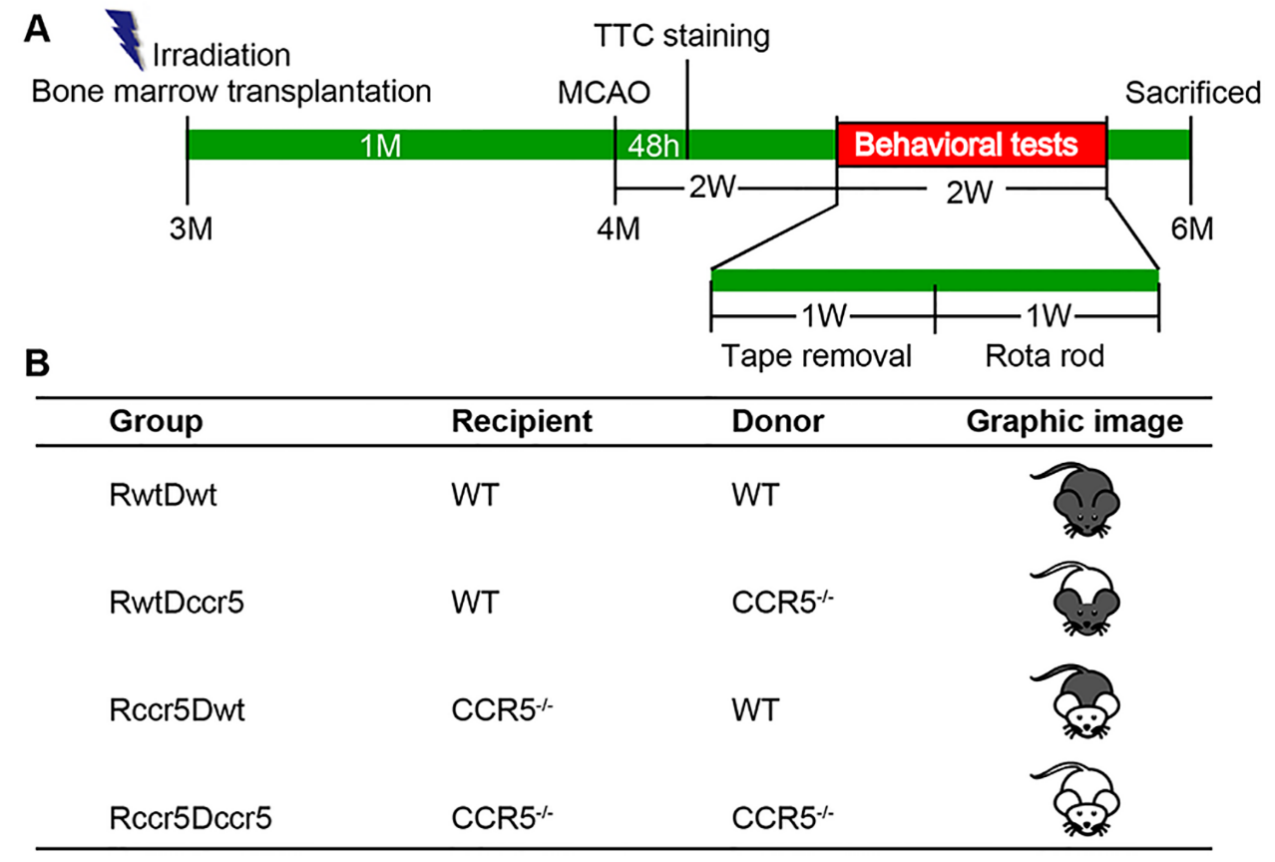
Experimental flowchart and groups. (A) A schematic diagram shows the flowchart of the experiment. Three-month-old wild type (WT) and CCR5^-/-^ mice received irradiation and bone marrow transplantation. After one month-bone marrow reconstruction, mice were subjected to middle cerebral artery occlusion (MCAO) for induction of focal cerebral ischemia. Triphenyl tetrazolium chloride (TTC) staining was performed 48h after MCAO to measure the infarction size (N = 6/group). Two weeks after MCAO, behavioral tests were performed to evaluate neurological deficits (N=9/group). Two months after MCAO, mice were sacrificed for immunohistochemistry (N=7-8/group). (B) A table shows the information about the four experimental groups of chimeric mice. RwtDwt: WT mice received bone marrow transplantation (BMT) from the bone marrow donor of WT mice. RwtDccr5: WT mice received BMT from the bone marrow donor of CCR5^-/-^ mice. Rccr5Dwt: CCR5^-/-^ mice received BMT from the bone marrow donor of WT mice. Rccr5Dccr5: CCR5^-/-^ mice received BMT from the bone marrow donor of CCR5^-/-^ mice.

## MATERIALS AND METHODS

The experimental procedures were conducted in accordance with the National Institutes of Health Guide for the Care and Use of Laboratory Animals in the United States. The research protocol of this study was approved by the Institutional Animal Care and Use Committee.

### Animals and experimental design

The original breeders of CCR5 knockout mice (CCR5^-/-^) were purchased from the Jackson Laboratory. The mouse colony was maintained by homozygous breeding. Age-matched C57BL/6J mice (Jackson Lab, Bar Harbor, USA) were used as wild type (WT) controls. Twenty-eight male WT mice were randomly divided into two groups, and the same random procedure was also used for grouping twenty-eight male CCR5^-/-^ mice (N=14/group). At the age of 3 months, the four groups of mice received a lethal dose (9.5 Gy, 950rad) of radiation to destroy the bone marrow stem cells. For head protection, mice were placed individually into a conical tube, and the head was covered by 8-cm thick lead shielding. The bone marrow (BM) from either WT or CCR5^-/-^ mice was then transplanted to the irradiated mice. Four groups of chimeric mice were used for this study: (1) irradiated WT mice transplanted with WT-BM, (2) irradiated WT mice transplanted with CCR5^-/-^-BM, (3) irradiated CCR5^-/-^ mice transplanted with WT-BM, and (4) irradiated CCR5^-/-^ mice transplanted with CCR5^-/-^-BM. After hematopoietic system reconstruction for 4 weeks, all the mice were subjected to focal cerebral cortical ischemia (stroke) in the right hemisphere. Forty-eight hours after brain ischemia, six mice from each group were randomly chosen for determining infarction size through brain slides stained with 2, 3, 5-Triphenyltetrazolium chloride (TTC, Sigma, St. Louis, MO, USA). The rest of the mice were subjected to a battery of behavioral tests two weeks after brain ischemia to examine neurological deficits. All mice were sacrificed two months after brain ischemia, and brain samples were collected for further histological and immunohistochemical analyses. An experimental flowchart (Fig. 1A) and experimental groups (Fig. 1B) are shown in Figure 1.

### Bone marrow transplantation and chimera generation

Donor mice, including both WT and CCR5^-/-^ mice (3 months old, male), were anesthetized with Avertin (Tribromoethanol) (0.4 g/kg body weight, i.p., Sigma, St. Louis, MO, USA). The femur and tibia were removed from the mice. After clipping the ends of the bones, BM cells were flushed out twice with 10ml ice-cold Hanks balanced salt solution (HBSS, Thermo Fisher Scientific, Waltham, MA, USA) through a 5ml-syringe with a 25-gauge needle. The collected BM cells were then filtered through a 70-μm nylon mesh (Thermo Fisher Scientific, Waltham, MA, USA), centrifuged and resuspended with HBSS into single cell suspension. Within 24h after irradiation, isolated BM cells were transplanted to the recipient mice by tail vein injection (1×10^7^ cells in 0.7 ml HBSS per mouse). The healthy status of the recipient mice was carefully monitored during the first week after transplantation.

### Animal model of stroke

Focal cerebral cortical ischemia was performed by permanent occlusion of both the right common carotid artery (CCA) and the right middle cerebral artery (MCA). Mice were anesthetized with Avertin and placed in a supine position on a heating pad. A rectal probe was inserted into the mice, and the body temperature of the mice was monitored and maintained between 37±0.5 using a temperature control system (FHC Inc., Bowdoin, ME, USA). After shaving the fur on the ventral neck region and disinfecting the surgical site, a middle line incision on the neck was made under a microscope. The right CCA was exposed, isolated and then ligated with a 3-0 silk surgical suture. After the incision on the neck was sutured, mice were changed to a lateral position, and the right-side head was exposed. The incision was made vertically between the right eye and ear. A craniotomy (1mm in diameter) was made using a high-speed micro-drill to expose the MCA. The MCA was occluded by electrocoagulation using a small cauterizer (Bovie^R^ Aaron medical, clearwater, FL, USA) without damaging the brain tissue.

### TTC staining

Forty-eight hours after the induction of brain ischemia, mouse brains were collected for TTC staining to evaluate the infarct volume. Mice were anesthetized with Avertin and euthanized by cervical dislocation. The brains were rapidly removed and sliced coronally into five 2mm-thick slices using a mouse brain matrix. The brain slices were stained in 2% pre-warmed TTC solution prepared in phosphate-buffered saline (PBS, Thermo Fisher Scientific, Waltham, MA, USA) for 20min in a 37 °C water bath. After staining, the brain slices were fixed in 10% neutral buffered formalin solution (Sigma, St. Louis, MO, USA) at 4 °C for 24h. The images of TTC-stained brain slices were captured with a Canon camera (PowerShot SX40 HS). The areas of contralesional (A_C_) and non-ischemic region in the ipsilesional hemisphere (A_Ni_) were measured by using NIH ImageJ software. The volume of contralesional (V_C_) and non-ischemic ipsilesional hemisphere (V_Ni_) was calculated following the formula: V_C_ or V_Ni_ = A_C_ or A_Ni_ × 2 mm (thickness of brain slice). To avoid the influence of cerebral edema, the infarction in each slice was normalized to the contralesional side. The infarction size was expressed as the percentage of infarct volume (see the following formula): Infarcted volume (%) = (∑ (V_C_ - V_Ni_ ) / ∑ V_C_) ×100.

### Behavioral tests

#### Tape removal test

The tape removal test, which evaluates somatosensory-motor function, was performed for two consecutive days. On the first day of the trial, the mice were placed in the testing container for 120 s to habituate the environment. The testing container is a transparent flat bottom beaker (20 cm in diameter). On the second day, two equal sized circular stickers (5mm in diameter) were attached to the bilateral forepaws using a pair of small forceps. The stickers were gently pressed on both sides of forepaws with the forceps. Mice were then placed in the testing beaker for 120 s. If the mouse removes the tapes from their forepaws before 120 s, the removal time of each tape is recorded through a video camera. If the mouse fails to remove one or both tapes, the trial is ended at 120 s, and the removal time is recorded as 120 s. The stickers were removed manually by the experimenter after 120 s. Each mouse received a total of 3 trials. Individual trial was separated from ~30 min resting time.

#### Rotarod test

The rotarod test, which evaluates the motor coordination function, was performed daily for five days. Each daily trial was composed of 3 independent trials with 30-minute intervals between each trial. Each day before starting the experiment, mice were placed in the testing room for 30 minutes to acclimate to the environment. Mice were placed on the rotarod machine facing away from the experimenter. Each mouse started on the rotating rod at 0 rotation per minute (rpm). The rod was then accelerated steadily from 2 rpm to 20 rpm during a 5-minute testing period (by 2 rpm every 30 seconds). The latency to fall on the floor plate was recorded.

### Tissue preparation

Two months after brain ischemia, mice were anesthetized with Avertin and then sacrificed through transcardiac perfusion of PBS containing heparin (10 U/ml, Sagent Pharmaceuticals, Schaumburg, IL, USA) followed by 10% neutral buffered formalin. After perfusion, brains were removed, post-fixed in the same fixative solution overnight at 4 °C, and then dehydrated in 30% sucrose solution (Sigma, St. Louis, MO, USA) in PBS for two days at 4 °C for cryoprotection. After being fully dehydrated, brains were embedded into optimal cutting temperature embedding medium (O.C.T. compound, Thermo Fisher Scientific, Waltham, MA, USA) on dry ice. Coronal brain sections with 30μm thickness were prepared using a Cryostat (Leica, Wetzlar, Germany). The brain sections were then stored in anti-freeze buffer (30% ethylene glycol, Sigma, and 30% glycerol, Sigma, in PBS) in -20 °C for further experiments.

### Cresyl violet staining

To measure the infarct volume, brain sections were stained with cresyl violet. Serial coronal sections at 30 μm thickness from the whole brain with 180 μm intervals were collected and washed with PBS. The brain sections were then mounted onto Superfrost plus slices (Thermo Fisher Scientific, Waltham, MA, USA). After fully air-dried, brain sections were stained in 0.1% cresyl violet solution (prepared with distilled water and 0.3% acetic acid, Sigma, St. Louis, MO, USA) for 10 min at room temperature. After rinsing in distilled water, brain sections were further differentiated in 95% ethanol (Warner Graham Company, Cockeysville, MD, USA) for 10 min followed by dehydrating in 100% ethanol (Warner Graham Company, Cockeysville, MD, USA). Finally, the sections were cleared in xylene (Thermo Fisher Scientific, Waltham, MA, USA) and mounted with Permount mounting medium (Thermo Fisher Scientific, Waltham, MA, USA). Whole brain sections were imaged by a microscope (Nikon, Tokyo, Japan). Using the images of cresyl violet-stained brain sections, the areas of contralesional (A_C_) hemisphere and ipsilesional remaining tissue (A_ir_) were measured by ImageJ software. The volume of contralesional (V_C_) and ipsilesional remaining (V_ir_) hemisphere was calculated following the formula: V_C_ or V_ir_ = A_C_ or A_ir_ × 210 μm (section thickness plus interval of brain sections). The infarction in each section was normalized to the contralesional side and expressed as the percentage of infarct volume. The total percentage of infarct volume was calculated with the following formula: [∑ (V_C_ - V_ir_) / ∑ V_C_] ×100.

### Immunohistochemistry

Three brain sections for each brain across the infarct cavities (bregma 1.34 to -1.06 mm) were randomly selected for immunohistochemistry. Briefly, brain sections were rinsed with PBS. Nonspecific binding was blocked with 10% donkey serum (Gemini bio-products, West Sacramento, CA, USA) prepared in 1% bovine serum albumin (BSA, IgG free, Jackson Laboratory, Bar Harbor, ME, USA) and 0.3% TritonX-100 (Sigma, St. Louis, MO, USA) solution for 1h at room temperature. For the primary antibodies from the mouse origin, the brain sections were further blocked with mouse-on-mouse blocking reagent (M.O.M ^TM^, Vectorshield, Burlingame, CA, USA) for 1h at room temperature. Sections were then incubated with primary antibodies overnight at 4°C. The primary antibodies used in this study were mouse anti-neuronal nuclei (NeuN, 1:1000, Chemicon, Burlington, MA, USA, cat. no. mab377), rabbit anti-microtubule associated protein 2 (MAP2, 1:600, Millipore, Burlington, MA, USA, cat. no. ab5622), mouse anti-post synaptic density protein 95 (PSD-95, 1:500, Novus, Saint Charles, MO, USA, cat. no. nbp2-12872), goat anti-ionized calcium-binding adapter molecule 1 (Iba-1, 1:500, Novus, Saint Charles, MO, USA, cat. no. nb100-1028), rabbit anti-purinergic receptor P2Y (P2RY12, 1:500, Brigham Health, Boston, MA, USA), rat anti-CD68 (1:500, Bio-rad, Hercules, CA, USA), rabbit anti-interleukin 4 (IL-4, 1:300, Thermo fisher Scientific, Waltham, MA, USA, cat. no. pa5-25165), rabbit anti-nitric oxide synthase 2 (NOS2, 1:300, rabbit, Millipore, Burlington, MA, USA, cat. no. ab5382), mouse anti-2',3'-Cyclic-nucleotide 3'-phosphodiesterase (CNPase, 1:300, Abcam, Cambridge, UK, cat. no. ab6319), rabbit anti-myelin basic protein (MBP, 1:500, Abcam, Cambridge, UK, cat. no. ab40390) and mouse anti-glial fibrillary acidic protein (GFAP, 1:500, Sigma, St. Louis, MO, USA, cat. no. g3893). The following day, brain sections were washed three times with PBS and then incubated with appropriate secondary antibodies for 2h at room temperature in the dark. The secondary antibodies used were Alexa Fluor 594- conjugated donkey anti-mouse (1:500, Thermo Fisher Scientific, Waltham, MA, USA, cat. no. a21203), Alexa Fluor 488- conjugated donkey anti- mouse (1:500, Thermo Fisher Scientific, Waltham, MA, USA, cat. no. a21202), Alexa Fluor 488- conjugated donkey anti-goat (1:500, Thermo Fisher Scientific, Waltham, MA, USA, cat. no. a11055), Alexa Fluor 594- conjugated donkey anti-rabbit (1:500, Thermo Fisher Scientific, Waltham, MA, USA, cat. no. a21207) and Alexa Fluor 594- conjugated donkey anti-rat (1:500, Thermo Fisher Scientific, Waltham, MA, USA, cat. no. a21209). To visualize the brain vessels, sections were incubated with Dylight 488-labeled Lycopersicon esculentum (Tomato) Lectin (1:1000, Vector, Burlingame, CA, USA, cat. no. dl-1174) for 2h at room temperature. The nuclei were counterstained by Hochest 33342 (1:10000, Sigma, St. Louis, MO, USA), and the sections were mounted by antifade mounting medium (Vectorshield, Burlingame, CA, USA). Negative control of immunostaining was performed by omission of the primary antibody.

### Fluoro-Jade C staining

To identify the degenerating neurons after brain ischemia, brain sections were processed for Fluoro-Jade C staining. Two brain sections for each brain across the infarct cavities (Bregma 1.34 to -1.06 mm) were randomly selected. After rinsing with PBS, brain sections were mounted onto the Superfrost plus slides and air-dried at room temperature overnight. After fully dried, slides were immersed in 100% ethanol for 3 min, followed with 70% ethanol for 1 min, and then rinsed in distilled water for 2 min. Next, the slides were incubated in 0.6% potassium permanganate (KMnO4, Sigma, St. Louis, MO, USA) solution for 15 min with gently shaking and then rinsed with distilled water for 2 min. The brain sections were then stained in 0.001% Fluoro-Jade C (Millipore, Burlington, MA, USA) solution prepared by 0.1% acetic acid (Sigma, St. Louis, MO, USA) for 30 min, and washed three times in distilled water for 2 min each time. The brain sections were further air-dried at room temperature overnight in the dark. The next day, sections were cleared with xylene and mounted with Permount mounting medium.

### Quantitative image analysis

Images were taken by a Zeiss 780 confocal microscope. Structural alterations were measured in the peri-infarct cortex (within 500 μm from the border of infarct cavity) [25]. For NeuN and Lectin staining, images were captured under a 10× lens. One field (400 × 400 μm^2^/field) adjacent to the infarct cavity was scanned for each section in the ipsilateral hemisphere. For Fluoro-Jade C staining, images were captured under a 20× lens. Two field (200 × 200 μm^2^/field) adjacent to the infarct cavity was scanned for each section in the ipsilateral hemisphere. The number of positive cells of NeuN and Fluoro-Jade C, and the positive area of Lectin were calculated by ImageJ (NIH software). For detecting MAP2 and PSD-95 positive staining, images were captured under a 40× lens with 1 μm-interval Z-stacks. Two fields (200 × 600 μm^2^/field) adjacent to the infarct cavity were scanned for each section in the ipsilateral hemisphere. After reconstructing into 3-dimensional images, the areas of MAP2 and PSD-95 immunopositive staining in layer1 and layer 2/3 were measured separately by ImageJ for statistical analysis. For other immunostaining including Iba1, P2RY12, CD68, IL-4, NOS2, CNPase, MBP and GFAP, two fields (200 × 200 μm^2^/field) next to the infarct cavity were scanned for each section in the ipsilateral hemisphere. The positive area of staining was calculated by ImageJ (NIH software).

### Statistical analysis

Data collection and analysis were carried out by using randomization and blind approaches. Depending on the distribution of the data, if the data fits the normal distribution, statistics were applied by one-way analysis of variance (ANOVA) with Tukey *post hoc* test or two-way ANOVA with Fisher’s LSD multiple comparisons. Correlation tests were calculated by using linear regression. Results were considered statistically significant when a p value was less than 0.05. Data were presented as mean ± standard error of mean (SEM). Statistical analysis was performed using Graphpad Prism (GraphPad Software, Inc., La Jolla, CA, USA).

**Figure 2.**
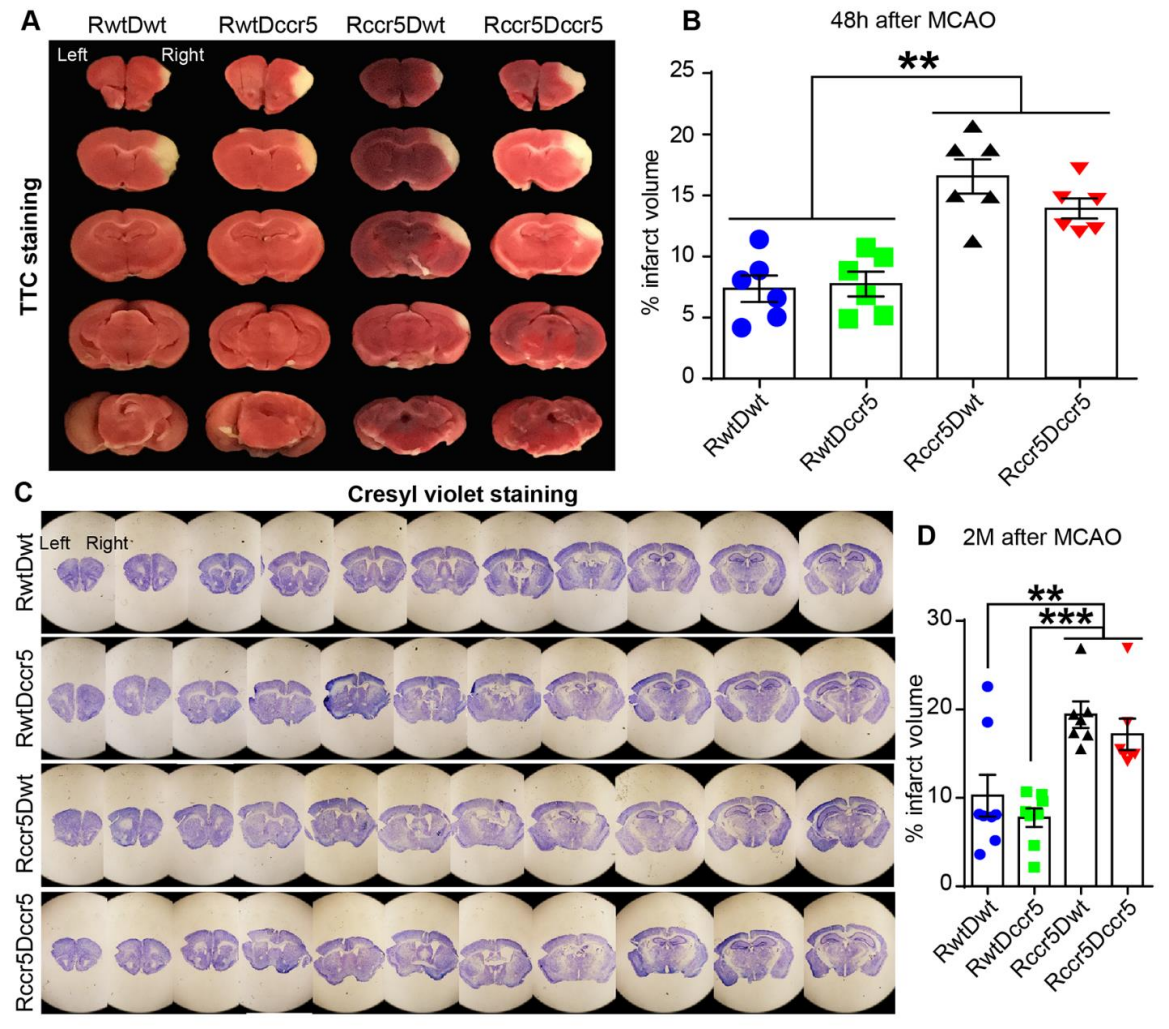
Infarct volume is increased in the mice lacking CCR5 in the brain. (A) Representative images of triphenyl tetrazolium chloride (TTC) staining 48h after middle cerebral artery occlusion (MCAO). (B) Quantification data of infarct volume using TTC staining. (C) Representative images of cresyl violet staining two months after MCAO. A longitudinal notch was made in the left brain to distinguish the contralesional hemisphere from the ipsilesional hemisphere. (D) Quantification data of infarct volume using cresyl violet staining. Mean ± S.E.M. N=6/group. **p<0.01, ***p<0.001. One-way ANOVA with Tukey *post hoc* test.

## RESULTS

### Brain-derived CCR5 is required for neuroprotection after cerebral cortical ischemia

Previous studies have shown the protective role of CCR5 in reducing brain injuries after cerebral ischemia in CCR5^-/-^ mice [22]. To further determine whether brain-derived CCR5 or bone marrow-derived CCR5 plays a key role in reducing brain damage after stroke, we performed bone marrow transplantation (BMT) in both WT and CCR5^-/-^ mice. Four groups of bone marrow chimeric mice were created: (1) WT mice with WT-BMT (RwtDwt), (2) WT mice with CCR5^-/-^ -BMT (RwtDccr5), (3) CCR5^-/-^ mice with WT-BMT (Rccr5Dwt), and (4) CCR5^-/-^ mice with CCR5^-/-^ -BMT (Rccr5Dccr5) (Fig. 1B). One month after BMT, these mice were subjected to focal cerebral ischemia. In order to elucidate the effects of brain-derived CCR5 or bone marrow-derived CCR5 on brain damage, we measured and analyzed infarct volume in the four groups at both the early stage (48h) and the late stage (2 months) after cerebral cortical ischemia.

The infarct volume data collected from TTC staining 48h after MCAO (Fig. 2A and B) showed that the infarct volume was significantly larger in Rccr5Dwt mice than that of RwtDwt mice and RwtDccr5 mice (p<0.01, Fig. 1B). We also found that the infarct volume was significantly increased in Rccr5Dccr5 mice as compared to the RwtDwt mice and the mice in the RwtDccr5 group (p<0.01, Fig. 1B). However, no statistical difference was found between RwtDwt and RwtDccr5 groups, or between Rccr5Dwt and Rccr5Dccr5 groups, suggesting that bone marrow-derived CCR5 deficiency does not affect brain damage after stroke. In addition, our data also reveal that the lack of CCR5 in the brain (Rccr5Dwt and Rccr5Dccr5 groups vs. RwtDwt and RwtDccr5 groups) leads to severe acute brain injury after experimental stroke. These findings indicate that brain-derived CCR5 plays a key role in neuroprotection under the pathological condition of focal cerebral ischemia.

Next, we examined the effects of both bone marrow-derived and brain-derived CCR5 on brain lesion 2 months after focal cerebral ischemia. Infarct volume was measured through serial coronal brain sections using cresyl violet staining (Fig. 2C). Similar to the findings of the TTC staining at 48h post-brain ischemia, the increased infarct volume was observed in Rccr5Dwt and Rccr5Dccr5 mice as compared to the mice in RwtDwt and RwtDccr5 groups (Rccr5Dwt and Rccr5Dccr5 mice vs. RwtDwt mice, p<0.01; Rccr5Dwt and Rccr5Dccr5 mice vs. RwtDccr5 mice, p<0.001; Fig. 2D). Again, no statistical difference was found between RwtDwt and RwtDccr5 groups, or between Rccr5Dwt and Rccr5Dccr5 groups. These findings show the same profile as seen at the early stage of ischemic stroke (48h post-MCAO), suggesting that at the late stage of ischemic stroke (2 months post-MCAO), brain derived-CCR5, but not the bone marrow-derived CCR5, is critically involved in neuroprotection after focal cerebral ischemia.

### Brain-derived CCR5 deficiency exaggerates brain ischemia-induced neurological deficits

To evaluate focal brain ischemia-caused somatosensory-motor functional deficits, we performed an adhesive tape removal test and a rotarod test.

**Figure 3.**
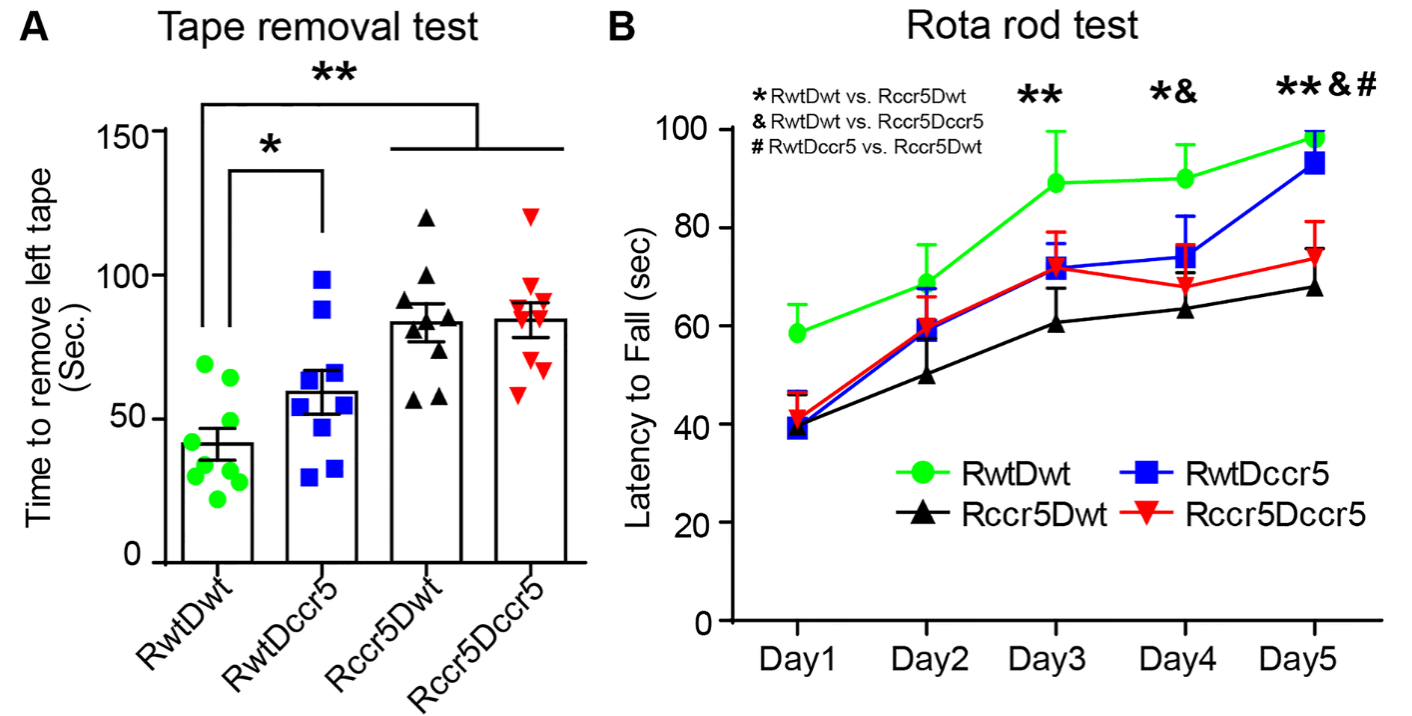
Cerebral cortical ischemia-induced neurological deficits are exacerbated in the mice lacking CCR5 in the brain. (A) Somatosensory-motor function was examined using a tape removal test. Data were collected from three independent trials each day. Mean ± S.E.M. N=9/group. *p<0.05, **p<0.01. One-way ANOVA with Tukey *post hoc* test. (B) Motor coordination function was examined using a rotarod test. Data were collected from three independent trials each day for five consecutive days. Mean± S.E.M. N=9/group. *p<0.05, **p<0.01; RwtDwt mice vs. Rccr5Dwt mice. &p<0.05, RwtDwt mice vs. Rccr5Dccr5 mice. #p<0.05, RwtDccr5 mice vs. Rccr5Dwt mice. Two-way repeated ANOVA with Fisher’s LSD multiple comparison test.

The tape removal test was performed two weeks after MCAO (Fig. 1A). As the cerebral cortical ischemia was induced in the right hemisphere, we measured and analyzed the latency to remove the adhesive tape on the left paw (i.e. the affected paw). We observed that there was a significantly longer time to remove the tape from the left paw in Rccr5Dwt mice than that of RwtDwt mice (p<0.01, Fig. 3A). We also found that the latency to remove the tape from the left paw was significantly increased in Rccr5Dccr5 mice as compared to the RwtDwt mice (p<0.01, Fig. 3A). However, no statistical difference was showed between RwtDccr5 and Rccr5Dccr5 mice, or between Rccr5Dwt and Rccr5Dccr5 groups (Fig. 3A). In addition, significantly increased time to remove the tape from the left paw was seen in RwtDccr5 mice as compared to the mice in RwtDwt group (p<0.05, Fig. 3A). Taken together, these data suggest that both brain- and bone marrow-derived CCR5 deficiency exaggerate brain ischemia-induced somatosensory-motor function impairments.

The rotarod test was performed three weeks after MCAO (Fig. 1A). On the first day test, a trend toward decreased latency to fall was found in RwtDccr5, Rccr5Dwt and Rccr5Dccr5 mice as compared to RwtDwt mice (RwtDccr5 vs. RwtDwt, p=0.06; Rccr5Dwt vs. RwtDwt, p=0.07; Rccr5Dccr5 vs. RwtDwt, p=0.09. Fig. 3B), suggesting possible impairments of motor coordination in the CCR5 deficiency mice. On Day 3 testing, RwtDwt mice showed significantly longer latency to fall than those from Rccr5Dwt group (p<0.01, Fig. 3B). On Day 4 and Day 5 testing, significantly increased latency to fall was also observed in RwtDwt mice when compared to the mice in both the Rccr5Dwt and Rccr5Dccr5 groups (Day 4: p<0.05. Day 5: RwtDwt mice vs. Rccr5Dwt mice, p<0.01; RwtDwt mice vs. Rccr5Dccr5, p<0.05. Fig. 3B). In addition, data of Day 5 testing also revealed a significant increase of latency to fall in RwtDccr5 mice as compared to Rccr5Dwt mice (p<0.05, Fig. 3B). No difference was found between RwtDwt mice and RwtDccr5 during the 5-day testing. Collectively, these findings suggest that brain-derived CCR5 deficiency exaggerates brain ischemia-impaired motor coordination.

### Brain-derived CCR5 inhibits neural degeneration after cerebral cortical ischemia

As stated earlier, our data demonstrated a protective role of brain-derived CCR5 in reducing infarct volume and neurological deficits after cerebral cortical ischemia. Since neurodegeneration and the integrity of neural structures plays an important role in brain functioning, we sought to determine the effects of CCR5 on neurodegeneration and neurostructural alterations 2 months after brain ischemia.

**Figure 4.**
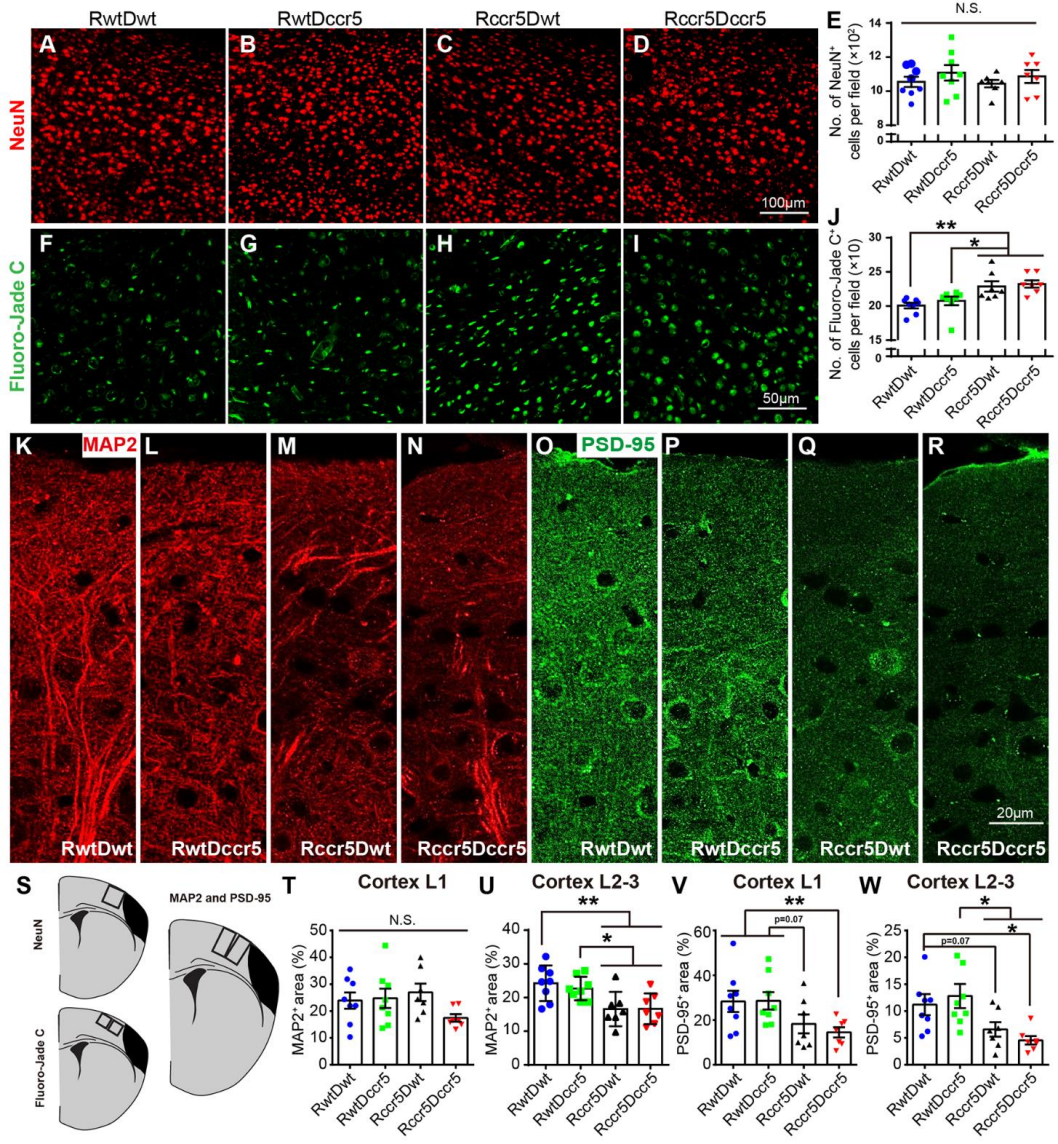
Neuronal degeneration in the peri-infarct cortex 2 months after cerebral cortical ischemia. Note that the mice lacking CCR5 in the brain show increased Fluoro-Jade C^+^ degenerating neurons, reduced MAP2^+^ dendritic density and decreased PSD-95^+^ post-synapses in the peri-infarct cortex. (A-D) Representative images show immunofluorescence staining for NeuN in the peri-infarct cortex. (E) Quantification data of NeuN positive neurons in the peri-infarct cortex. (F-I) Representative images show Fluoro-Jade C staining in the peri-infarct cortex. (J) Quantification data of Fluoro-Jade C positive degenerating neurons in the peri-infarct cortex. (K-R) Representative images show MAP2^+^ dendrites and PSD-95^+^ post-synapses in the peri-infarct cortical layer 1-3. (S) Schematic diagrams indicate the imaging areas in the cortex. (T-W) Quantification data of MAP2 and PSD-95 positive area in the peri-infarct cortical layer 1 and layer 2/3. Mean± S.E.M. N=7-8/group. *p<0.05, **p<0.01. One-way ANOVA with Tukey *post hoc* test. N.S.: not significant.

We first examined neuronal density in the peri-infarct cortex through NeuN immunohistochemistry. NeuN is a universally specific marker for mature neurons [26]. We found that there was no significant difference in NeuN^+^ neurons among the four experimental groups in the peri-infarct cortex (Fig. 4A-E). However, using Fluoro-Jade C staining, which identifies degenerating neurons [27], we observed that both the Rccr5Dwt mice and Rccr5Dccr5 mice showed significant increases of Fluoro-Jade C^+^ cells in the peri-infarct cortex as compared to the RwtDwt mice and the RwtDccr5 mice (Rccr5Dwt and Rccr5Dccr5 mice vs. RwtDwt mice, p<0.01; Rccr5Dwt and Rccr5Dccr5 mice vs. RwtDccr5 mice p<0.05. Fig. 4F-J). There was no significant difference seen in Fluoro-Jade C^+^ cells between RwtDwt and RwtDccr5 mice, or between Rccr5Dwt and Rccr5Dccr5 mice (Fig. 4F-J). These data suggest that brain-derived CCR5, but not bone marrow-derived CCR5, is required for inhibiting neurodegeneration in the peri-infarct cavity cortex after cortical ischemia. The findings of Fluoro-Jade C^+^ degenerative neurons are not in line with the findings of NeuN^+^ neurons in the peri-infarct cortex, suggesting that the pathological status of neurodegeneration has not yet reached the levels to kill neurons.

Next, we further examined dendritic densities in the peri-infarct cortex through MAP2 immunostaining. MAP2 is one of the most prominent proteins concentrated in the dendrites of neurons and involved in regulating dendrite growth [28, 29]. MAP2 is also widely used as a neuronal marker [30]. We observed that MAP2 positive dendrites did not show significant difference in cortical layer 1 of peri-infarct cortex among the four experimental groups (Fig. 4K-N and T). However, MAP2 positive densities in cortical layer 2/3 of peri-infarct area were significantly reduced in both the Rccr5Dwt and Rccr5Dccr5 mice as compared to the mice in RwtDwt and RwtDccr5 groups (Rccr5Dwt and Rccr5Dccr5 mice vs. RwtDwt mice, p<0.01; Rccr5Dwt and Rccr5Dccr5 mice vs. RwtDccr5 mice, p<0.05. Fig. 4K-N and U). No significant difference was observed between RwtDccr5 group and RwtDwt group, or between Rccr5Dwt and Rccr5Dccr5 mice (Fig. 4K-N and U). These data confirm that brain-derived CCR5 plays a protective role in inhibiting neural structural damage in the peri-infarct cavity cortex after brain ischemia.

We then wanted to know whether CCR5-associated synaptic alterations also occur in the peri-infarct cortex. To this end, we examined synaptic density in the peri-infarct cortex 2 months after brain ischemia by PSD-95 immunostaining. PSD-95, the most abundant protein in the postsynaptic density area [31], is the major scaffolding protein located at excitatory synapses and is involved in the stabilization, recruitment and trafficking of neurotransmitters to the postsynaptic membrane [32]. As presented in Figure 4, PSD-95 showed similar expression profiles in both the peri-infarct cortical layer 1 and layer 2/3 in the experimental groups. In cortical layer 1 and layer 2/3, PSD-95^+^ area was significantly reduced in Rccr5Dccr5 group as compared to both the RwtDwt and RwtDccr5 groups (Cortical layer 1: RwtDwt and RwtDccr5 vs. Rccr5Dccr5, p<0.01; Cortical layer 2/3: RwtDwt and RwtDccr5 vs. Rccr5Dccr5, p<0.05. Fig. 4O-R, V and W), revealing that lacking CCR5 in the brain exacerbates the loss of PSD-95^+^ post-synapses particularly under the condition of lacking CCR5 in the bone marrow. The PSD-95^+^ area in cortical layer 2/3 was also significantly reduced in Rccr5Dwt mice in comparison with RwtDccr5 mice (p<0.05. Fig. 4O-R, and W). No significant difference was seen between RwtDwt and RwtDccr5 groups, or between Rccr5Dwt and Rccr5Dccr5 groups (Fig. 4O-R, V and W), suggesting that bone marrow-derived CCR5 is not responsible for losing PSD-95^+^ post-synapses. We also observed that there was a trend toward decreasing of PSD-95^+^ area in Rccr5Dwt mice as compared to RwtDwt mice (p=0.07) in both the cortical layer 1 and layer 2/3. These data suggest that brain-derived CCR5 is needed for preventing PSD-95^+^ synapse loss after brain ischemia.

To determine the interaction between infarct volume, neural degeneration and neurological deficits, correlation tests were performed (Supplementary Fig. 1A-J). Since the most significant differences among all experimental groups were seen on Day 5 of rotarod testing, the data of Day 5 testing were selected for the correlation analysis. Our data revealed that the neurodegeneration (Fluoro-Jade C positive cells) in the peri-infarct cortex was significantly and positively correlated with the infarction size (p<0.05, Supplementary Fig. 1A) and tape removal latency (p<0.05, Supplementary Fig. 1D). We also observed a significantly negative correlation between dendritic density in the peri-infarct cortex and infarction size (p<0.05, Supplementary Fig. 1B), between the dendritic density in the peri-infarct cortex and tape removal latency (p<0.01, Supplementary Fig. 1E), or between synaptic density in the peri-infarct cortex and infarction size (p<0.01, Supplementary Fig. 1C). Only a trend toward negative correlation was found between synaptic density in the peri-infarct cortex and tape removal latency (p=0.06, Supplementary Fig. 1F). However, neurodegeneration, dendritic density, and synaptic density in the peri-infarct cortex were not correlated with latency to fall of rotarod Day 5 testing (p > 0.05, Supplementary Fig. 1G-J). These findings suggest that there is a tight connection between infarct volume and neural/neurostructural degeneration in the peri-infarct cortex, and between neural/neurostructural degeneration in the peri-infarct cortex and somatosensory-motor function impairments. The increased infarction size leads to increases of neurodegeneration, dendritic loss, and synaptic loss in the peri-infarct cortex, resulting in more severe impairments of somatosensory-motor function (adhesive tape removal test). Motor coordination (rotarod test) impairment after cerebral cortical infarct appears not to have tight correlation with the degenerative neural structures in the peri-infarct cortex.

### Brain-derived CCR5 deficiency modulates long-term neuroinflammation after cerebral cortical ischemia

Cerebral ischemia leads to local immune responses including microglial cell accumulation and monocyte-derived macrophage infiltration from the circulation [33]. We then sought to investigate the inflammatory cell accumulation in a CCR5 deficiency condition after brain ischemia. In this study, we examined Iba1, P2RY12 and CD68 expressing cells in the peri-infarct cortex using immunohistochemistry 2 months after MCAO. Among these markers, Iba1 is the pan marker for microglia and macrophages. Expression of Iba1 has been reported to be up-regulated in the brain after ischemia [34]. P2RY12 is a specific marker for the resident microglial cells at homeostatic status in the CNS of healthy rodents, and it can distinguish microglial cells from blood-borne macrophages [35]. CD68 is the marker commonly used for identifying macrophages and activated microglial cells [36]. To determine ischemic brain lesion-induced long-term neuroinflammation, intact mice were used as naïve controls

**Figure 5.**
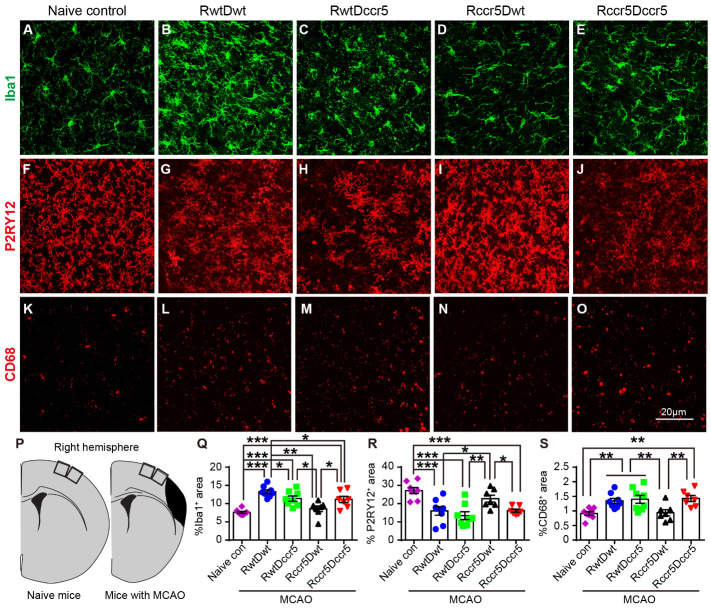
Inflammatory cells in the peri-infarct cortex 2 months after cerebral cortical ischemia. Note that reduced Iba1^+^ cells, increased P2RY12^+^ cells and decreased CD68^+^ cells in the peri-infarct cortex are seen in the mice lacking CCR5 in the brain (Rccr5Dwt). (A-E) Representative images of immunofluorescence staining for Iba1 in the peri-infarct cortex. (F-J) Representative images of immunofluorescence staining for P2RY12 in the peri-infarct cortex. (K-O) Representative images of immunofluorescence staining for CD68 in the peri-infarct cortex. (P) Schematic diagrams show the imaging areas in the cortex. (Q-S) Quantification data of Iba1, P2RY12 and CD68 positive area in the peri-infarct cortex. Mean± S.E.M. N=7-8/group. *p<0.05, **p<0.01, *** p<0.001. One-way ANOVA with Tukey *post hoc* test.

We observed that the area of Iba1^+^ cells was significantly increased in the peri-infarct cortex of RwtDwt, RwtDccr5 and Rccr5Dccr5 mice as compared to the naïve mice at 2 months post-ischemia (p<0.001, Fig. 5A-C, E and Q), suggesting that cortical ischemia causes a long-term inflammatory response in the peri-infarct cortex in these mice. However, no statistical difference was found between the naïve control group and Rccr5Dwt ischemic group (Fig. 5A, D and Q). In addition, increased Iba1^+^ expressing cells were found in the peri-infarct cortex of RwtDwt mice as compared to the Rccr5Dwt mice (p<0.01, Fig 5B, D and Q). These findings suggest that the brain-derived CCR5 deficiency inhibits ischemia-induced long-term neuroinflammation.

Moreover, a significant decrease of Iba1^+^ cells in the peri-infarct cortex was observed in RwtDccr5 mice as compared to RwtDwt group (p<0.05, Fig. 5B, C and Q), suggesting that the lack of bone marrow-derived CCR5 also reduces peri-infarct cortical long-term neuroinflammation. However, Rccr5Dwt mice showed significant reduction of Iba1^+^ cells in the peri-infarct cortex when compared to the RwtDccr5 mice (p<0.05, Fig. 5C, D and Q), indicating brain-derived CCR5 deficiency has more robust effects in reducing long-term peri-infarct cortical neuroinflammation than that of bone marrow-derived CCR5 deficiency. In addition, we also noted that Rccr5Dccr5 mice showed significant increases of Iba1^+^ cells in the peri-infarct cortex in comparison with Rccr5Dwt mice (p<0.05, Fig. 5D, E and Q), while no difference was found between RwtDccr5 mice and Rccr5Dccr5 mice. This observation suggests that the robust effects of brain-derived CCR5 deficiency in reducing Iba1^+^ cells in the peri-infarct cortex may be interactive with the bone marrow-derived CCR5. This finding is confirmed by the data of CD68^+^ cells (Fig. 5S).

The data of P2RY12^+^ homeostatic microglia reveal an expression pattern that is the opposite to the data of Iba1^+^ cells in the peri-infarct cortex. P2RY12^+^ homeostatic microglia in the peri-infarct cortex were significantly reduced in the ischemic mice in the groups of RwtDwt, RwtDccr5 and Rccr5Dccr5 as compared to the naïve mice at 2 months post-ischemia (p<0.001, Fig. 5F-H, J and R). These data suggest that reduced homeostatic microglia in the peri-infarct cortex of these mice. We did not observe statistical difference between the naïve control group and Rccr5Dwt ischemic group (Fig. 5R). Moreover, the P2RY12^+^ homeostatic microglia were significantly increased in the Rccr5Dwt mice as compared to the RwtDwt mice (p<0.05), RwtDccr5 mice (p<0.01) and Rccr5Dccr5 mice (p<0.05) (Fig. 5R). No statistical difference of P2RY12^+^ cells was found between RwtDwt mice and RwtDccr5 (Fig. 5R). These findings suggest that the brain-derived CCR5 deficiency, but not the bone marrow-derived CCR5 deficiency, plays a key role in maintaining homeostatic microglia and reducing the ischemia-built long-term inflammatory microenvironment in the peri-infarct cortex. This effect of brain-derived CCR5 deficiency may also interact with the bone marrow-derived CCR5.

In line with our findings of Iba1 and P2RY12 expressing cells, CD68^+^ cells in the peri-infarct cortex were also significantly increased in RwtDwt, RwtDccr5 and Rccr5Dccr5 mice as compared to the naïve mice at 2 months post-ischemia (p<0.01, Fig. 5K-M, O and S). This observation further confirms that long-term neuroinflammation exists in the peri-infarct cortex. Again, no difference in CD68^+^ cells was found between the naïve controls and Rccr5Dwt ischemic mice (Fig. 5S). The CD68^+^ cells in the peri-infarct cortex were significantly reduced in Rccr5Dwt mice when compared to RwtDwt mice, RwtDccr5 mice and Rccr5Dccr5 mice (p<0.01) (Fig. 5S). There was no difference in the CD68^+^ cells between the RwtDwt mice and RwtDccr5 (Fig. 5S). These findings further validate our observation in P2RY12 and Iba1 expressing cells and indicate the efficacy of brain-derived CCR5 deficiency, but not the bone marrow-derived CCR5 deficiency, in reducing post-ischemic long-term neuroinflammation in the peri-infarct cortex. The bone marrow-derived CCR5 may play an assistive role to the brain-derived CCR5 deficiency in reducing the long-lasting neuroinflammation in the peri-infarct cortex.

To find out the interactive relationship between peri-infarct cortical long-term neuroinflammation and infarction size, and between peri-infarct cortical long-term neuroinflammation and neurological deficits, we performed correlation tests. Surprisingly, our data showed that the infarction size was negatively correlated with the Iba1 expressing cells in the peri-infarct cortex (p<0.05, Supplementary Fig. 2A). However, the infarction size had no correlation with either P2RY12 or CD68 positive cells in the peri-infarct cortex (Supplementary Fig. 2B and C). This observation suggests a critical involvement of Iba1^+^ cells in reducing the brain lesion at the late stage of cerebral cortical stroke. In addition, we also observed a significantly *negative* correlation between tape removal latency and Iba1^+^ cells in the peri-infarct cortex (p<0.05, Supplementary Fig. 2D) and a significantly *positive* correlation between latency-to-fall in rotarod day 5 test and Iba1^+^ cells in the peri-infarct cortex (p<0.05, Supplementary Fig. 2G), suggesting that Iba1^+^ cells in the peri-infarct cortex play a key role in improving somatosensory-motor functional outcomes. Neither P2RY12 nor CD68 expressing cells in the peri-infarct cortex showed statistical correlation with neurological deficits (Supplementary Fig. 2E-F and H-I).

As neuroinflammation results in neural damage, we then further examined the correlation between neuroinflammation and neural damage in the peri-infarct cortex. To our surprise, our data showed that there was no statistical correlation between Iba1^+^ cells and any of the Fluoro-Jade C^+^ degenerating neurons, MAP2^+^ dendrites or PSD-95^+^ synapses in the peri-infarct cortex (Supplementary Fig. 2J-L). These correlation analyses reveal a novel supportive role of peri-infarct cortical Iba1^+^ cells in infarction size reduction and functional improvement in the subacute phase of cortical ischemia.

### CCR5 deficiency increases inflammatory molecule production

**Figure 6.**
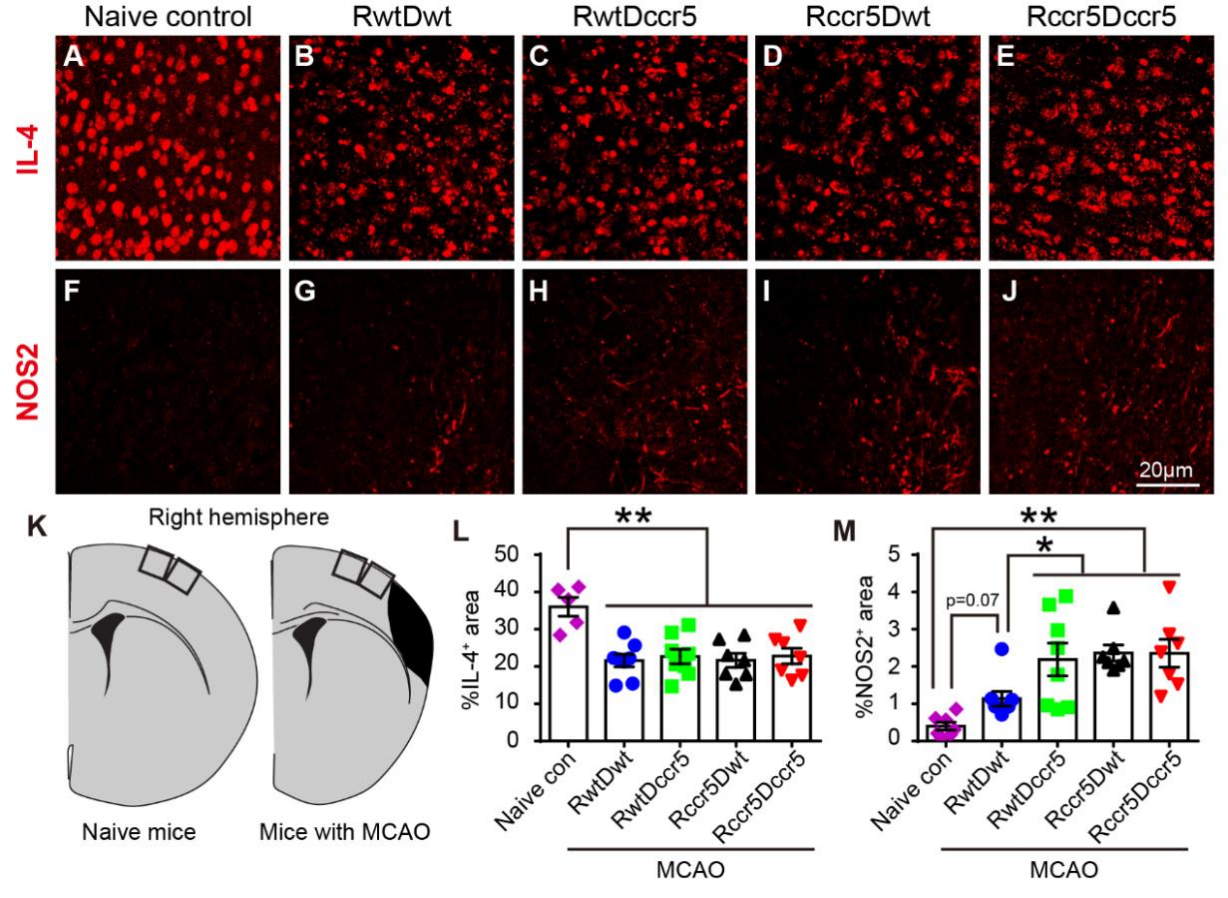
Neuroinflammatory molecule expression in the peri-infarct cortex 2 months after cerebral cortical ischemia. Note that stroke mice show reduced IL-4 expression and increased NOS2 expression in the peri-infarct cortex as compared to the naïve control mice. Stroke mice lacking CCR5 in both the brain and bone marrow show increased NOS2 expression in the peri-infarct cortex (RwtDccr5, Rccr5Dwt, and Rccr5Dccr5 vs. RwtDwt). (A-E) Representative images of immunofluorescence staining for IL-4 in the peri-infarct cortex. (F to J) Representative images of immunofluorescence staining for NOS2 in the peri-infarct cortex. (K) Schematic diagrams indicate the imaging areas in the cortex. (L and M) Quantification data of IL-4 and NOS2 positive area in the peri-infarct cortex. Mean± S.E.M. N=7-8/group. *p<0.05, **p<0.01. One-way ANOVA with Tukey *post hoc* test.

To determine the molecular basis of the neuroinflammation in CCR5 deficient mice, we examined the expression of IL-4 and NOS2 in the peri-infarct cortex. IL-4 is a pleiotropic cytokine involved in the regulation of diverse immune and inflammatory responses. It has been demonstrated that IL-4 activates macrophages and promotes phagocytosis-mediated tissue clean up, resulting in improved functional recovery [37]. NOS2 is also a key regulator in the post-ischemic inflammatory cascade. It has been reported that inhibiting NOS2 leads to a long-lasting protective effect in brain injury [38], implying that NOS2 acts as a pro-inflammatory molecule. Our data showed that there was no statistical difference of IL-4 expression among the four ischemic groups (Fig. 6A-E and L). However, the expression of NOS2 in the peri-infarct cortex was significantly increased in RwtDccr5 mice, Rccr5Dwt mice and Rccr5Dccr5 mice as compared to RwtDwt mice (p<0.05, Fig. 6G-J and M) and naïve control mice (p<0.01, Fig. 6F, H-J and M). A trend toward increased NOS2 expression in the peri-infarct cortex was seen in RwtDwt mice as compared to naïve control mice (p=0.07, Fig. 6F, G and M). No difference was observed among the RwtDccr5 mice, Rccr5Dwt mice and Rccr5Dccr5 mice. These findings suggest that both brain-derived CCR5 and bone marrow-derived CCR5 are crucially involved in chronic neuroinflammation 2 months after MCAO.

Correlation analysis did not show significant correlation between peri-infarct cortical NOS2 and infarction size, or between peri-infarct cortical NOS2 and neurological deficits (Supplementary Fig. 3). These data indicate that brain-derived CCR5 deficiency-induced increases of infarction size and exaggeration of neurological deficits are not related with chronic neuroinflammation in the peri-infarct cortex.

### Brain-derived CCR5 deficiency reduces myelination in the peri-infarct cortex after cerebral cortical ischemia

**Figure 7.**
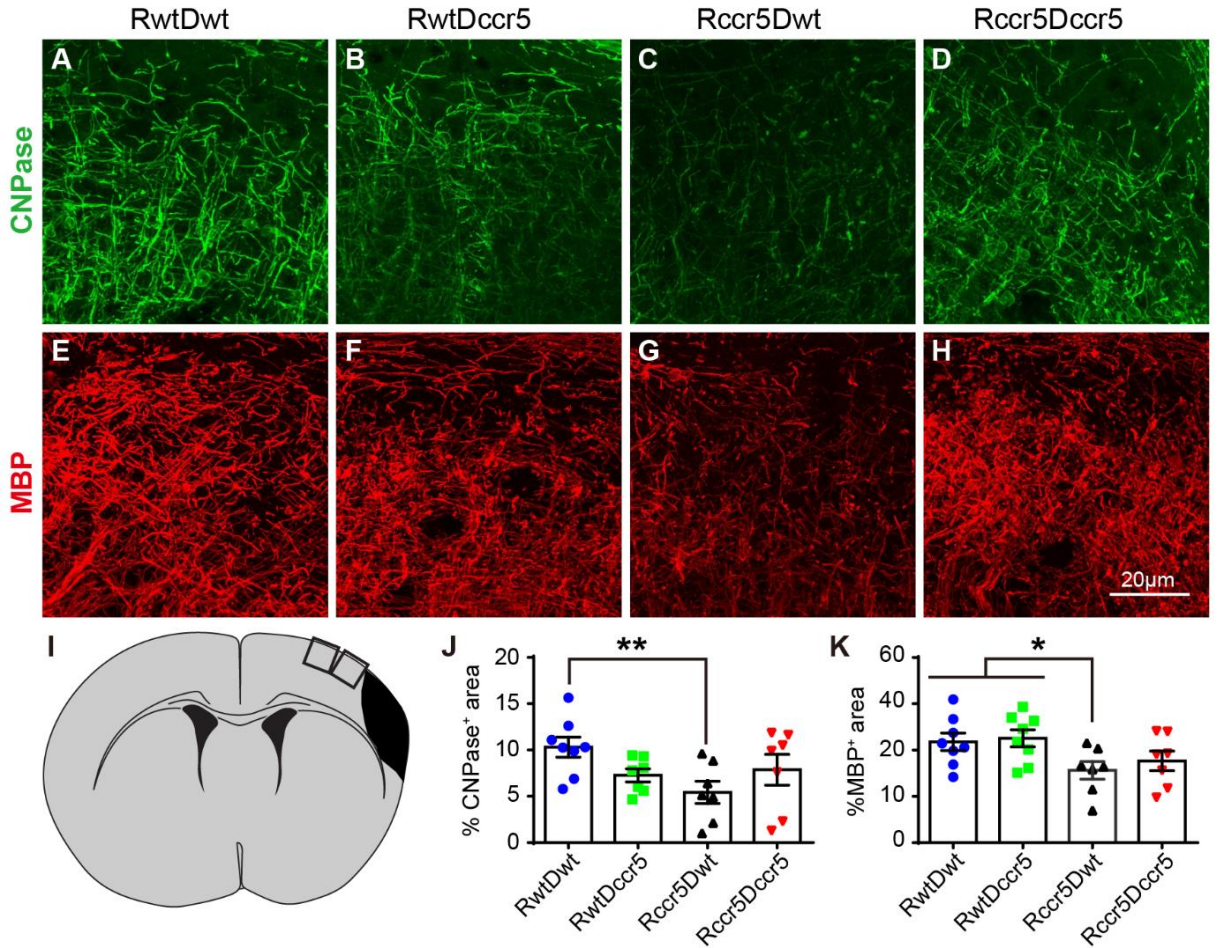
Myelination in the peri-infarct cortex 2 months after cerebral cortical ischemia. Note that mice lacking CCR5 in the brain show reduced myelination in the peri-infarct cortex 2 months after cerebral cortical ischemia. (A-D) Representative images of immunofluorescence staining for CNPase in the peri-infarct cortex. (E-H) Representative images of immunofluorescence staining for MBP in the peri-infarct cortex. (I) A schematic diagram indicates the imaging areas in the cortex. (J and K) Quantification data of CNPase and MBP positive areas in the peri-infarct cortex. Mean± S.E.M. N=7-8/group. *p<0.05, **p<0.01. One-way ANOVA with Tukey *post hoc* test.

Next, we sought to determine whether CCR5 deficiency influences myelination after brain ischemia. Myelin formation has been identified as a specific target of brain repair after stroke. Oligodendrocytes are responsible for initiating a cascade of events that results in the formation of myelin [39]. In the present study, we used CNPase, which is the marker for oligodendrocytes, and MBP to determine myelin formation by immunohistochemistry. We found that the percentage of CNPase expressing area in the peri-infarct cortex was significantly reduced only in the Rccr5Dwt mice when compared with RwtDwt mice (p<0.01, Fig. 7A-D and J), suggesting that brain-derived CCR5 is required for post-brain ischemia myelination in the axons of the peri-infarct cortex. Similar findings were seen in MBP immunostaining. The percentage of MBP^+^ area in the peri-infarct cortex was significantly decreased in Rccr5Dwt mice as compared to both the RwtDwt mice and RwtDccr5 mice (p<0.05, Fig. 7E-H and K). The expressing areas of CNPase and MBP did not show significant difference among the other groups (Fig. 7J and K). These data indicate again that brain-derived CCR5 deficiency impairs myelin formation in the peri-infarct cortex two months after MCAO.

Correlation tests showed significantly negative correlations between CNPase^+^ area and infarction severity (p<0.01, Supplementary Fig. 4A), and between CNPase^+^ area and tape removal latency (p<0.05, Supplementary Fig. 4C). These data suggest that oligodendrocytes in the peri-infarct cortex play a supportive role in reducing cortical infarction size and improving somatosensory-motor functional recovery at the late stage of experimental stroke. However, no significant correlation was found between CNPase^+^ area in the peri-infarct cortex and latency-to-fall in rotarod test (Supplementary Fig. 4D), suggesting that motor coordination is not related to the density of oligodendrocytes in the peri-infarct cortex.

MBP expression in the peri-infarct cortex did not show significant correlations with infarction size and neurological deficits (Supplementary Fig. 4B, E and F). Since myelin loss is associated with neuroinflammation [40], we also examined correlation between neuro-inflammation (Iba1 expressing area) and myelination (CNPase^+^ area and MBP^+^ area). We selected Iba1 expression for neuroinflammation because only Iba1 expression cells in the peri-infarct cortex showed significant correlation with the infarction size and neurological deficits. Surprisingly, we found a significantly positive correlation between Iba1 expression cells and CNPase^+^ oligodendrocytes in the peri-infarct cortex (p<0.05, Supplementary Fig. 4G). These data show that myelination in the peri-infarct cortex is associated with Iba1^+^ cells in the same area.

### Brain-derived CCR5 deficiency has no effects on vascular density in the peri-infarct cortex

**Figure 8.**
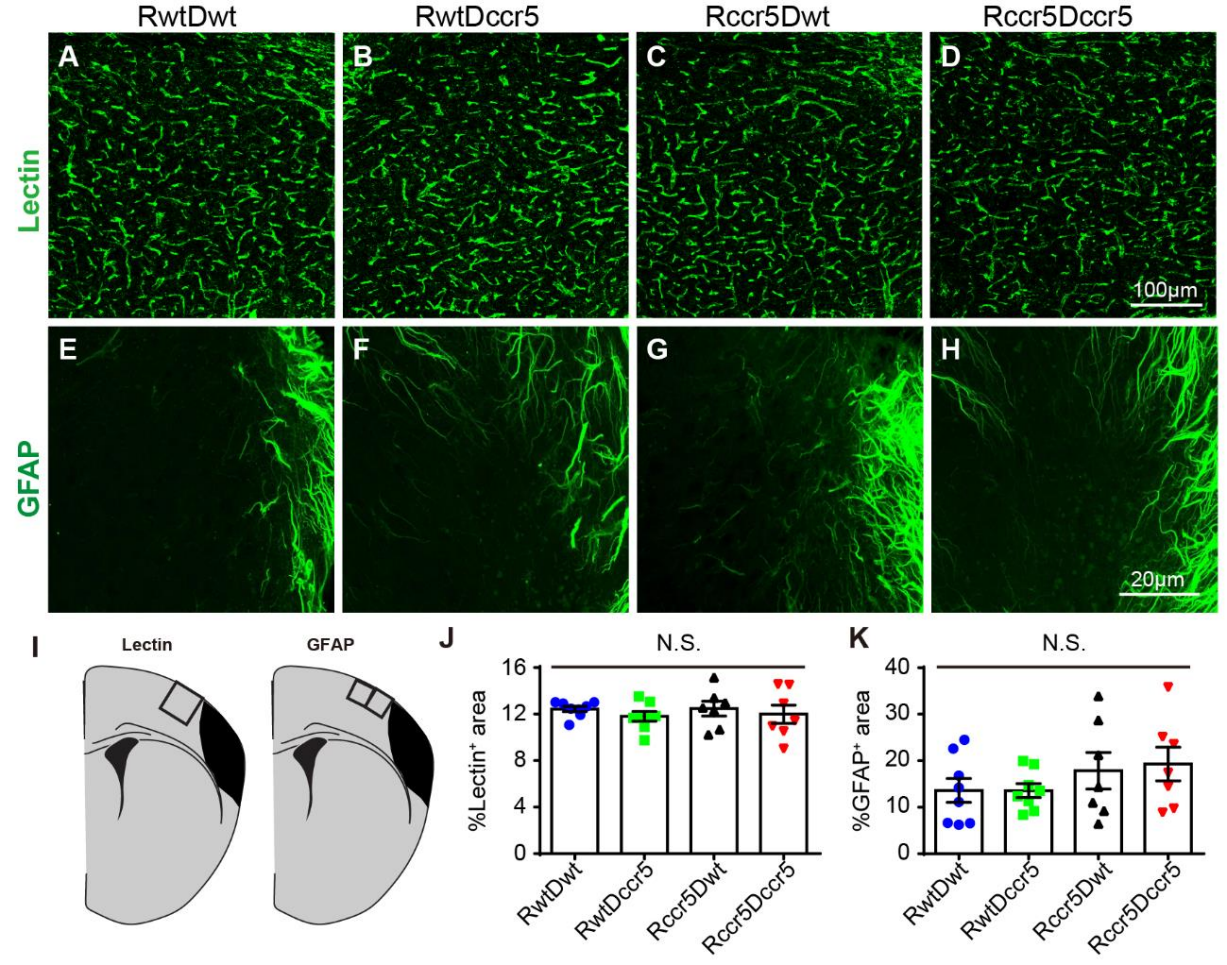
Blood vessel density and astrogliosis in the peri-infarct cortex are not affected by CCR5 deficiency. (A-D) Representative images show Lectin positive blood vessels in the peri-infarct cortex. (E-H) Representative images of immunofluorescence staining for GFAP in the peri-infarct cortex. (I) Schematic diagrams indicate the imaging areas in the cortex. (J and K) Quantification data of Lectin and GFAP positive area in the peri-infarct cortex. Mean ± S.E.M. N=7-8/group. One-way ANOVA with Tukey *post hoc* test. N.S.: not significant.

Finally, we examined whether CCR5 deficiency leads to the alteration of vascular density in the peri-infarct cortex two months after MCAO. It has been shown that post-stroke angiogenesis contributes to long-term recovery after stroke [41]. We assessed blood vessel density in the peri-infarct cortex using Lectin staining. We found that Lectin positive blood vessels in the peri-infarct cortex were not significantly different among the four experimental groups (Fig. 8A-D and J), suggesting that CCR5 deficiency has no effects on vascular changes after brain ischemia.

Astrocytes form astrogliosis to provide a physical barrier from the injury area and promote angiogenesis [42]. We therefore examined the astrocyte density in the peri-infarct cortex using GFAP immunostaining. Similar to the data of Lectin staining, GFAP positive cells in the peri-infarct cortex did not show significant difference among the four stroke groups (Fig. 8E-H and K). This observation shows that CCR5 deficiency does not affect astrogliosis in the peri-infarct cortex two months after induction of cortical ischemia.

## DISCUSSION

In this study we have identified the effects of brain-derived CCR5 or bone marrow-derived CCR5 on brain protection after cerebral cortical ischemia. Our data have revealed that after cerebral cortical ischemia: (1) Brain-derived CCR5, not bone marrow-derived CCR5, mainly contributes to neuroprotection in both acute and subacute phases; (2) Brain-derived CCR5 deficiency plays the predominant role of exacerbating neurological deficits in the subacute phase; (3) Brain-derived CCR5 shows protective and reparative effects on reducing neural degeneration, modulating neuroinflammation and promoting myelination in the subacute phase; (4) The protective and reparative effects of brain-derived CCR5 are significantly associated with both Iba1 positive cells and oligodendrocytes in the peri-infarct cortex.

### The role of CCR5 in neuroprotection after cerebral cortical ischemia

CCR5 is well known to be responsible for the chemotaxis of immune cells [15]. It has been reported that CCR5 is expressed in microglia, monocytes and macrophages [16, 17, 43]. It is well established that cerebral cortical ischemia leads to brain inflammation in which resident microglial activation and bone marrow-derived monocyte/macrophage infiltration to the ischemic hemisphere are involved [23, 44, 45]. In addition to participating in neuroinflammation, emerging evidence reveals that the infiltration of bone marrow-derived monocytes/macrophages plays an important role in brain repair after brain ischemia [7, 23]. It has been shown that the gene expressions of ccr5 and the ligands of ccr5 (ccl3, ccl4, and ccl5) are increased in the brain 24h and 7 days after MCAO, and that CCR5 knockout mice exacerbate the severity of cerebral infarction at 2 and 7 days post-MCAO and worsen motor function deficits 24h and 3 days after experimental stroke [22]. However, the mechanism underlying the neuroprotective effects of CCR5 after focal brain ischemia remains largely unknown. It is not clear whether brain-derived CCR5 or bone marrow-derived CCR5 contributes to protecting the brain from damage during cerebral focal ischemia. In the present study, we have demonstrated, for the first time, that brain-derived CCR5 is a key player in neuroprotection after cerebral cortical ischemia.

In this study, through the approach of bone marrow transplantation to generate bone marrow chimeric mice we have discovered that brain-derived CCR5 deficiency leads to severe cortical infarcts in both the acute (48 h) and subacute phases (2 months) of cerebral cortical ischemia. The lack of bone marrow-derived CCR5 has no effect in changing the infarction size. These findings indicate that brain-derived CCR5 is responsible for neuroprotection after cerebral cortical ischemia. The brain lesion data have been further confirmed by neurobehavioral tests. We have observed that brain-derived CCR5 deficient mice display exacerbated motor coordination deficits.

### The role of CCR5 in neurostructural network reorganization in the peri-infarct cortex

Numerous studies have demonstrated that neural network reorganization in the peri-infarct cortex is tightly related to functional improvement in both stroke patients and in experimental stroke [8, 9, 25, 46-48]. Clinical studies using functional magnetic resonance imaging have revealed that a progressive increase of activity in the ipsilateral somatosensory motor cortex occurs during functional recovery in stroke patients [46, 49]. Dynamic changes of dendritic density and dendritic spines in the peri-infarct cortex have been seen during the period of 6 weeks after cortical ischemia in mice [50]. Administration of stem cell factor (SCF) and granulocyte colony-stimulating factor (G-CSF) (SCF+G-CSF) has shown increases of dendritic density, dendritic spines, and synapses in the peri-infarct cortex and improvements of functional outcomes after experimental stroke [9, 25]. Blocking neurostructural and synaptic generation in the peri-infarct cortex leads to elimination of the SCF+G-CSF-improved functional recovery [8, 9]. In the current study, we have demonstrated the supportive effects of brain-derived CCR5 on enhancing neurostructure remodeling in the peri-infarct cortex in the subacute phase of experimental stroke (2 months post-MACO). We have also revealed that post-stroke neurodegeneration, and the alterations of dendritic density and synaptic density in the peri-infarct cortex are significantly correlated with the infarction size and neurological deficits.

Sorce and colleagues [22] reported that CCR5 deficient mice showed increases of apoptotic neuronal cell death in the infarct core 7 days after experimental stroke. In the present study, we have observed that brain-derived CCR5 deficient mice show increased degenerating neurons in the peri-infarct cortex at 2 months post-ischemia. In addition, dendritic density and synaptic density in the peri-infarct cortex are also reduced in the mice lacking CCR5 in the brain. These data indicate that brain-derived CCR5 is required for ameliorating neural degeneration and dendritic/synaptic loss in the peri-infarct cortex in the late subacute phase of cortical ischemia. By contrast to our findings, a recent study [19] has revealed that knocking down CCR5 in the neurons of pre-motor cortex in the ipsilateral hemisphere leads to increased dendritic spines in the ipsilateral pre-motor cortex, enhanced axonal sprouting in the contralateral cortex and improved functional outcomes. The discrepancies between our findings and Joy’s data suggest that the underlying mechanisms of brain-derived CCR5 in post-stroke neuronal connective network remodeling are very complex. The effects of brain-derived CCR5 in maintaining and modulating neurostructural connections after stroke may be in a cell type dependent manner (neurons vs. all cells expressing CCR5 in the brain).

Similar to our observations in the mouse model of cortical ischemic stroke, another study also reveals increased neurodegeneration and reduced dendritic density in the subacute phase of traumatic brain injury [27]. The increased neurodegeneration together with the reduced dendritic density and synaptic density could be a key pathological feature in the subacute phase of brain injury. These unique pathological changes may contribute to increased brain damage and impaired neurological function in the subacute phase of brain injury.

The correlation data of our study have revealed that brain infarction size has a positive correlation with peri-infarct cortical neurodegeneration, but a negative correlation with peri-infarct cortical dendritic density and synaptic density. Somatosensory-motor functional deficits with delayed time for tape removal from the affected paw also show a positive correlation with peri-infarct cortical neurodegeneration, but a negative correlation with peri-infarct cortical dendritic density and synaptic density. These findings indicate that infarction size-dependent neurodegeneration and dendritic/synaptic loss in the peri-infarct cortex play a key role in affecting somatosensory-motor functional recovery after cortical ischemia. To our surprise, there is no significant correlation between motor coordination deficits tested by rotarod and the neurodegeneration/neurostructure alterations in the peri-infarct cortex. The Rotarod test detects motor coordination function in rodents in which cerebellar function is also involved [51, 52]. The motor performance on the rotarod is not only related to the brain damage in the somatosensory-motor cortex, but it is also influenced by several other factors, such as motor coordination control, cerebellar dysfunction, and motor skill learning.

### The role of CCR5 in neuroinflammation in the subacute phase of cerebral cortical ischemia

Our data have revealed that brain-derived CCR5 deficiency leads to increased infarction size, increased neural degeneration, and decreased long-term inflammatory cell accumulation (Iba1^+^ cells and CD68^+^ cells) in the peri-infarct cortex 2 months after cortical ischemia. In the peri-infarct cortex, only the Iba1^+^ cells are associated with CCR5 deletion-related infarct volume changes and neurological deficits.

The role of CCR5-mediated inflammatory response under pathological conditions in the CNS has not been fully understood. Many studies have demonstrated that CCR5 expression is involved in increasing neuroinflammatory response in multiple sclerosis (MS) [53] and Alzheimer’s disease [54]. By contrast, only a few studies have explored the effects of CCR5 in stroke. In CCR5-deficient mice, a massive infiltration of neutrophils into the infarct core has been found 7 days after cerebral focal ischemia [22]. However, none of these studies have demonstrated whether CCR5 in peripheral immune cells or brain-derived CCR5 plays the neuroinflammatory role. The findings of the present study provide elaborate information that brain-derived CCR5 plays a key role in modulating long-term inflammatory cell accumulation in the peri-infarct cortex. P2RY12 is a specific marker for detecting the homeostatic/resting microglia in the brain [55-57]. P2RY12 is also a purinergic receptor which plays a key role in maintaining homeostatic microglia [56]. CD68 is the marker commonly used for identifying activated microglial cells [58]. It has been shown that at two weeks post-spinal cord injury, the activated microglia return to homeostasis with increased P2RY12 expression and decreased CD68 expression in the injury area [59]. Accordingly, the increased P2RY12 expression and decreased CD68 expression in the peri-infarct cortex of Rccr5Dwt mice demonstrate that the microglia homeostasis is recovered at 2 months post-cortical ischemia by knocking out CCR5 in the brain.

The brain-derived CCR5 deficiency-induced recovery of microglia homeostasis in the late subacute phase of stroke, however, does not support neuroprotection nor functional recovery. The data of our correlation tests have revealed that only Iba1^+^ cells, but not CD68^+^ cells or P2RY12^+^ cells, in the peri-infarct cavity cortex have significantly negative correlations with infarction size and somatosensory-motor functional impairments (tape removal test), and a significantly positive correlation with motor coordination function (rotarod test). These data suggest that Iba1^+^ cell accumulation in the peri-infarct cavity cortex is beneficial to infarction size reduction and functional recovery in the late subacute phase of experimental stroke.

Iba1 is expressed in both the resident microglia and monocyte-derived macrophages [60, 61]. Our findings have revealed that P2RY12-labled resident microglial cells are increased in the peri-infarct cavity cortex in mice lacking CCR5 in the brain (Rccr5Dwt mice), whereas these mice (Rccr5Dwt mice) also show increased infarction size and worsened functional outcomes. Notably, the correlation analysis data show that P2RY12^+^ cells in the peri-infarct cavity cortex have no correlation with either the infarction size or functional outcomes. These findings indicate that resident microglial cells in the peri-infarct cavity cortex are not involved in neuroprotection and functional recovery in the late subacute phase of experimental stroke. By contrast, Iba1^+^ macrophages in the peri-infarct cavity cortex may play a key role in protecting brain tissue loss during the subacute phase and in improving functional recovery in the subacute phase. This view is supported by recent studies revealing a pro-inflammatory role of monocyte-derived macrophages in the acute phase of experimental stroke in mice and an anti-inflammatory role of the macrophages in the subacute phase of focal brain ischemia in mice [23, 24]. The monocyte-derived macrophages gradually switch to anti-inflammatory phenotype 7 to 14 days after focal brain ischemia in mice [23, 24]. Blocking the infiltration of the monocyte-derived macrophages into the brain during the first week [23] or 0~14 days [24] after stroke in mice leads to increases of infarction size at 14 days post-stroke [24] and elimination of spontaneous functional recovery 2~11 weeks after stroke [23, 24].

It is not fully understood how macrophages protect against neuron loss and improve functional outcomes in the subacute phase of experimental stroke. In the present study, we have examined the expression of anti-inflammatory and pro-inflammatory molecules in the peri-infarct cavity cortex 2 months after stroke. We have found that the expression of the anti-inflammatory cytokine IL-4 is reduced in the peri-infarct cavity cortex of all stroke mice, which is not influenced by CCR5 knockout. Although the pro-inflammatory NOS2 expression in the peri-infarct cortex is increased in all stroke mice, the mice lacking CCR5 in the brain and/or bone marrow display increased expression of NOS2. These findings suggest that the cortical infarct-caused long-term neuroinflammation in the peri-infarct cortex is exacerbated by CCR5 knockout. NOS2 is generated by many types of cells including microglia, astrocytes and infiltrating immune cells [62]. Our correlation data, however, show that NOS2 expression in the peri-infarct cavity cortex is not correlated with infarction size and the severity of functional deficits. These findings suggest that anti-inflammation may not be the key mechanism underlying macrophage infiltration-induced functional recovery in the subacute phase of experimental stroke. It remains an open question for future studies to address how Iba1 expressing cells in the peri-infarct cavity cortex modulate somatosensory and motor functional improvement at the later stage of stroke.

### The role of CCR5 in brain demyelination in the subacute phase of cerebral cortical ischemia

The myelin sheath enwrapping axons ensures fast signal transmission [63, 64] and is comprised of oligodendrocyte processes. Myelination is largely dependent on oligodendrocytes [64]. Both clinical and basic science studies have revealed that stroke causes demyelination (loss of myelin sheath) in the axons of grey matter and white matter, resulting in long-term impairments of sensorimotor and cognitive functions [39, 65, 66].

The inflammatory role of CCR5 has been widely studied and recognized in CNS infection and MS [53, 67]. MS-induced neuroinflammation has been demonstrated to play a crucial role in demyelination [68]. However, the role of CCR5 in stroke-induced demyelination remains largely unknown.

It has been demonstrated that the increase of oligodendrocytes is an indicator of a cellular response for remyelination after brain injury [69, 70]. In the present study, we have determined axon myelination by quantifying CNPase^+^ oligodendrocytes and oligodendrocyte-produced MBP in the peri-infarct cortex 2 months after stroke. Our findings have revealed that both the CNPase^+^ oligodendrocytes and MBP-labeled myelin are significantly reduced in mice specifically lacking CCR5 in the brain, indicating that lack of brain-derived CCR5 exacerbates demyelination in the peri-infarct cortex in the late subacute phase of experimental stroke.

Our correlation analysis data show that the CNPase^+^ oligodendrocyte accumulation in the peri-infarct cortex is negatively correlated with both the infarction size and severity of somatosensory-motor function (tape removal latency), suggesting that oligodendrocyte accumulation in the peri-infarct cortex is an important event during the subacute phase of stroke for limiting brain tissue loss and improving functional recovery. Interestingly, we have also observed that oligodendrocyte accumulation in the peri-infarct cortex is positively correlated with Iba1^+^ cells in the same area. Accumulating evidence has revealed that infiltrated macrophages interact with oligodendrocytes and schwann cells to promote axon remyelination [71, 72]. The accumulated oligodendrocytes and Iba1^+^ macrophages in the peri-infarct cortex may play an important role in enhancing axon remyelination and improving functional recovery in the late subacute phase of stroke.

In conclusion, we have demonstrated that brain-derived CCR5 is critically involved in protecting the brain from post-ischemic damage, enhancing brain repair and improving functional recovery in the subacute phase after cerebral cortical ischemia. The cellular basis of these beneficial effects is related to the reduced neuronal degeneration, increased Iba1^+^ macrophages, increased oligodendrocytes and enhanced remyelination in the peri-infarct cortex. The limitation of this study that could be determined in future research is the lack of identifying which type of cells expressing CCR5 in the brain contribute to neuroprotection and neural repair after cerebral cortical ischemia. This study sheds new light on the protective and reparative role of CCR5 in the brain after stroke.

## Supplementary Materials

The Supplemenantry data can be found online at: www.aginganddisease.org/EN/10.14336/AD.2020.0406.


