# Supplementary material for: Brain-derived CCR5 Contributes to Neuroprotection and Brain Repair after Experimental Stroke
Source: Aging Dis. 2021 Feb 1;12(1):72–92. doi: 10.14336/AD.2020.0406 (PMC7801286; doi:10.14336/AD.2020.0406)
Supplement: Supplementary file 1 [file AD-12-1-72-s.pdf]

SUPPLEMENTARY DATA

Brain-derived CCR5 Contributes to Neuroprotection and Brain Repair after Experimental Stroke

Suning Ping, Xuecheng Qiu, Michele Kyle, Li-Ru Zhao*

© 2020. Ping S et al. Published online at http://www.aginganddisease.org/EN/10.14336/AD.2020.0406

SUPPLEMENTARY DATA

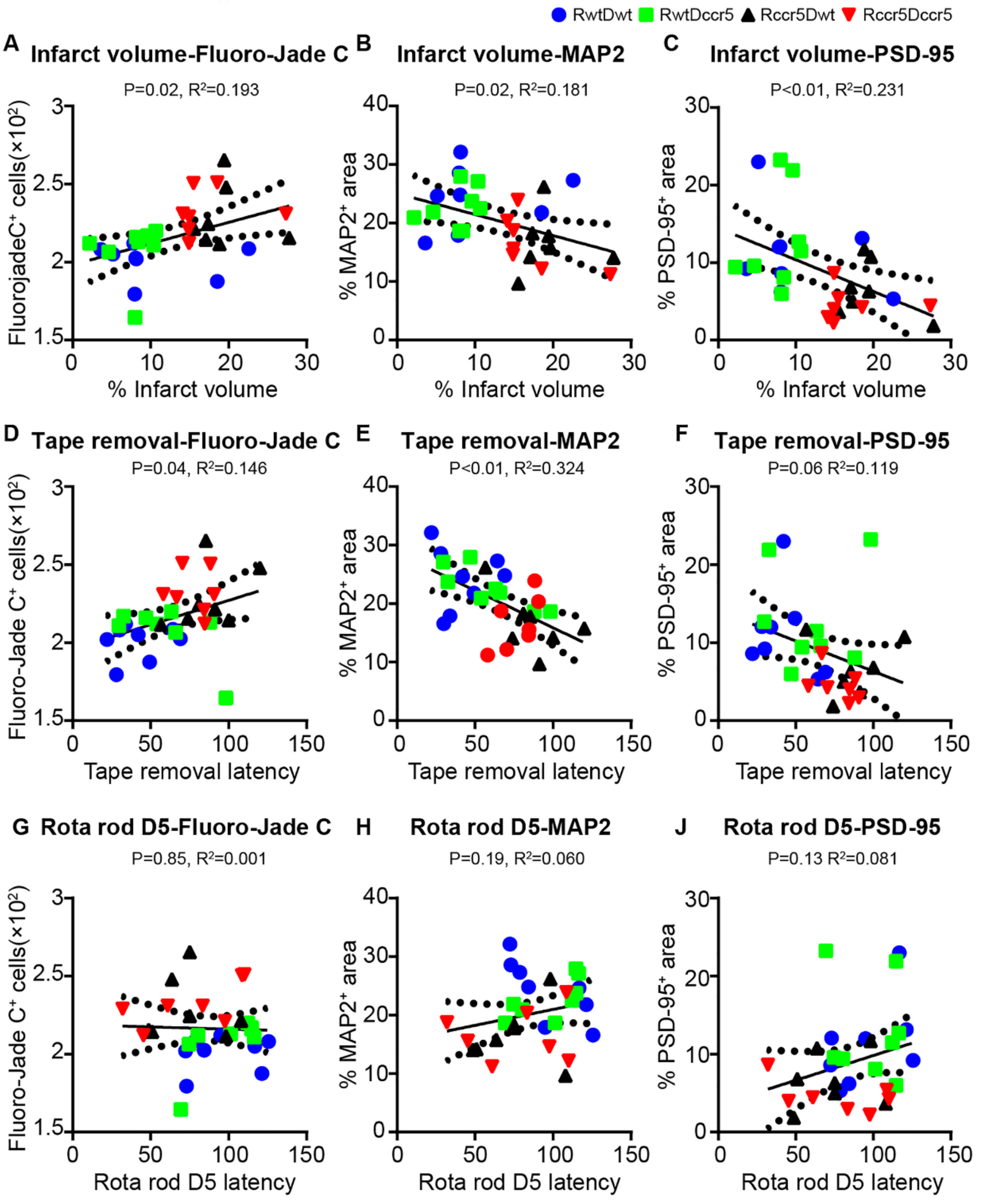

Supplementary Figure 1. Correlation analysis data. Note that Fluoro-Jade C positive degenerating neurons in the peri-infarct cortex show significantly positive correlation with infarct volume and latency to remove tape from the affected paw. The densities of $MAP2^+$ dendrites and $PSD\text{-}95^+$ post-synapses in the peri-infarct cortex are negatively correlated with infarct volume and the latency to remove tape from the affected paw. (**A**) Positive correlation between infarct volume and degenerating neurons (Fluoro-Jade C $^+$) in the peri-infarct cortex 2 months after MCAO. (**B and C**) Negative correlation between infarct volume and dendritic density ($MAP2^+$) (B) or between infarct volume and post-synapses ($PSD\text{-}95^+$) (C) in the peri-infarct cortex 2 months after MCAO. (**D**) Positive correlation between tape removal latency and neuron degeneration (Fluoro-Jade C $^+$) in the peri-infarct cortex. (**E and F**) Negative correlation between tape removal latency and dendritic density ($MAP2^+$) (E) or between tape removal latency and post-synapses ($PSD\text{-}95^+$) (F) in the peri-infarct cortex. (**G-J**) No correlation between the latency to fall at day 5 of rotarod test and neuron degeneration (Fluoro-Jade C $^+$) (G), dendritic density ($MAP2^+$) (H) or post-synapses ($PSD95^+$) (J) in the peri-infarct cortex.

© 2020. Ping S et al. Published online at http://www.aginganddisease.org/EN/10.14336/AD.2020.0406

SUPPLEMENTARY DATA

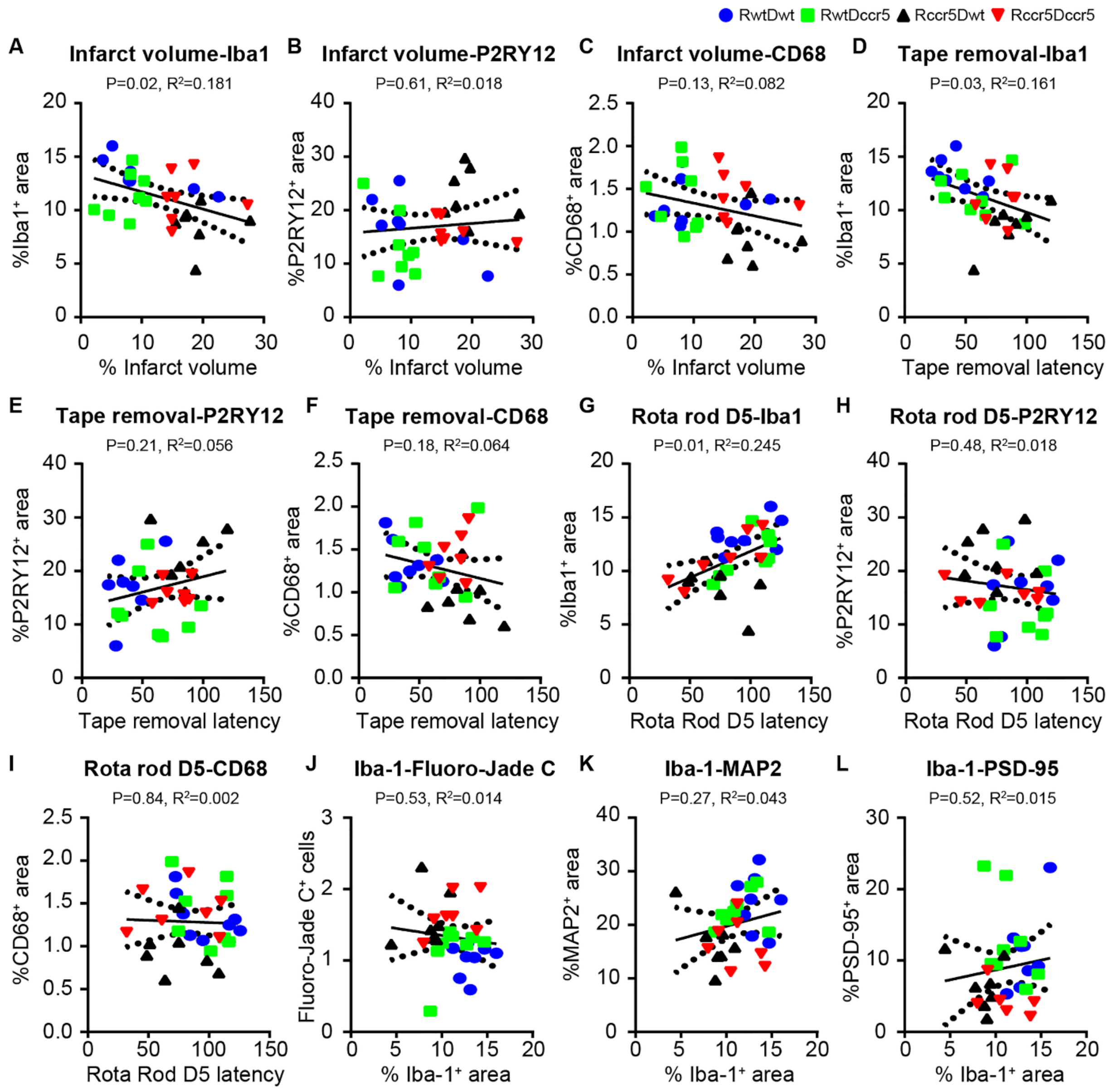

Supplementary Figure 2. Correlation analysis data. Note that Iba1 positive area shows significantly negative correlation with infarct volume (A) and the latency to remove tape from the affected paw (D), but significantly positive correlation with the latency to fall at day 5 of rotarod test (G). (**A**) Significantly negative correlation between infarct volume and $Iba1^+$ cells in the peri-infarct cortex 2 months after MCAO. (**B and C**) No correlation between infarct volume and $P2RY12^+$ cells (B), or between infarct volume and $CD68^+$ cells (C) in the peri-infarct cortex 2 months after MCAO. (**D**) Significantly negative correlation between tape removal latency and $Iba1^+$ cells in the peri-infarct cortex. (**E and F**) No correlation between tape removal latency and $P2RY12^+$ cells (E), between tape removal latency and $CD68^+$ cells (F) in the peri-infarct cortex. (**G**) Significantly positive correlation between the latency to fall at day 5 of rotarod test and $Iba1^+$ cells in the peri-infarct cortex. (**H and I**) No correlation between the latency to fall at day 5 of rotarod test and $P2RY12^+$ cells (H), or between the latency to fall at day 5 of rotarod test and $CD68^+$ cells (I) in the peri-infarct cortex. (**J-L**) No correlation between $Iba1^+$ cells and Fluoro-Jade C^+ degenerating neurons (J), between $Iba1^+$ cells and dendritic density ($MAP2^+$) (K), or between $Iba1^+$ cells and post-synapses ($PSD\text{-}95^+$) (L) in the peri-infarct cortex.

SUPPLEMENTARY DATA

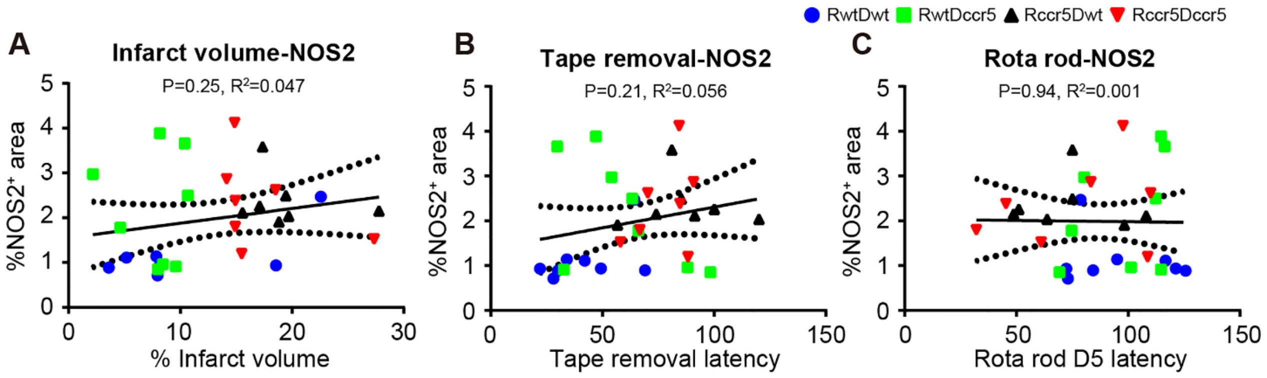

Supplementary Figure 3. Correlation analysis data. Note that NOS2 expression in the peri-infarct cortex shows no correlation with infarct volume (**A**), the latency to remove tape from the affected paw (**B**), and the latency to fall at day 5 of rotarod test (**C**).

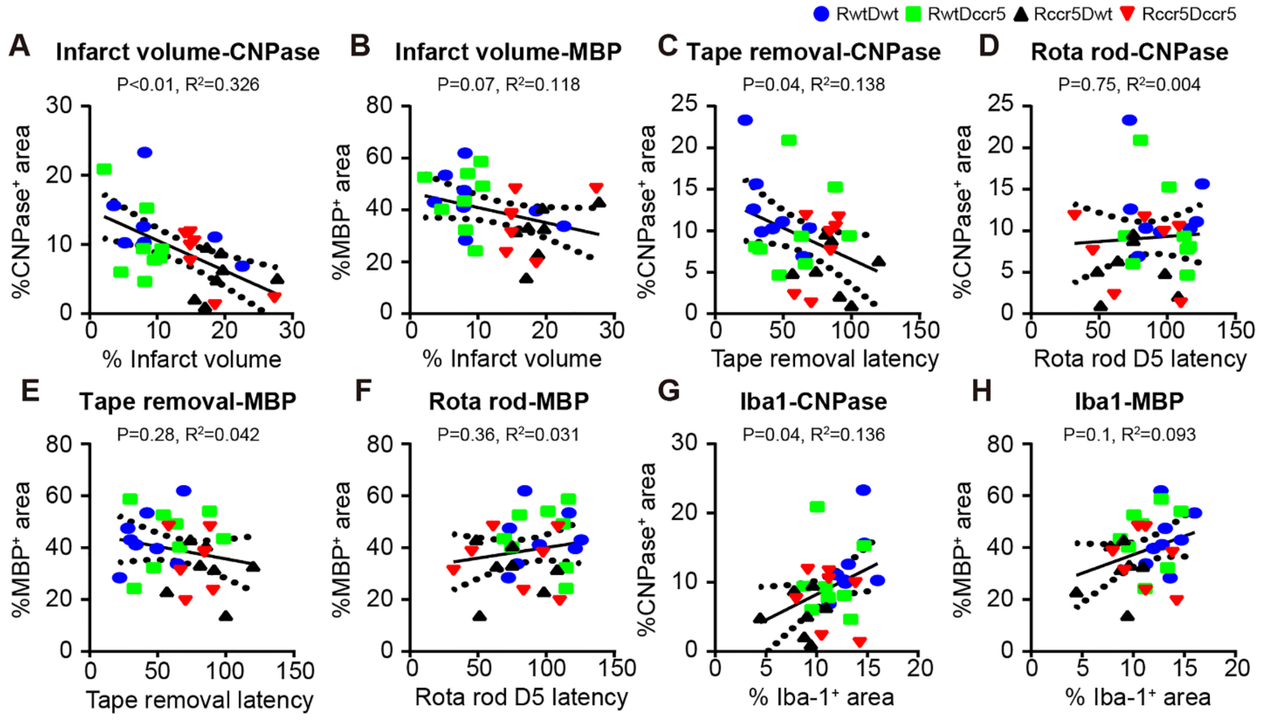

Supplementary Figure 4. Correlation analysis data. Note that the myelination (CNPase$^+$ oligodendrocytes and MBP$^+$ myelin sheath) in the peri-infarct cortex is negatively correlated with infarct volume and somatosensory-motor functional deficits. CNPase$^+$ oligodendrocyte area is also positively correlated with Iba1$^+$ cell area in the peri-infarct cortex. (**A**) Significantly negative correlation between infarct volume and CNPase$^+$ area in the peri-infarct cortex 2 months after cerebral cortical ischemia. (**B**) Negative correlation between infarct volume and MBP$^+$ area in the peri-infarct cortex. (**C**) Significantly negative correlation between tape removal latency and CNPase$^+$ area in the peri-infarct cortex. (**D**) No correlation between the latency to fall at day 5 of rotarod test and CNPase$^+$ area in the peri-infarct cortex. (**E and F**) No correlation between neurological deficits (tape removal test and rotarod test) and MBP$^+$ area in the peri-infarct cortex. (**G**) Significantly positive correlation between Iba1$^+$ area and CNPase$^+$ area in the peri-infarct cortex. (**H**) No correlation between Iba1$^+$ area and MBP$^+$ area in the peri-infarct cortex.

© 2020. Ping S et al. Published online at http://www.aginganddisease.org/EN/10.14336/AD.2020.0406
